# A New Family of Benzo[*h*]Chromene Based Azo Dye: Synthesis, In-Silico and DFT Studies with In Vitro Antimicrobial and Antiproliferative Assessment

**DOI:** 10.3390/ijms22062807

**Published:** 2021-03-10

**Authors:** Alaa S. Abd-El-Aziz, Azhaar Alsaggaf, Eman Assirey, Arshi Naqvi, Rawda M. Okasha, Tarek H. Afifi, Mohamed Hagar

**Affiliations:** 1 Department of Chemistry, University of Prince Edward Island, Charlottetown, PE C1A 4P3, Canada; zalsaggaf@yahoo.com (A.A.); eassirey@taibahu.edu.sa (E.A.); 2 Department of Chemistry, Taibah University, Madinah 30002, Saudi Arabia; arshi_84@yahoo.com (A.N.); rokasha@taibahu.edu.sa (R.M.O.); 3 Department of Chemistry, College of Sciences, Taibah University, Yanbu 30799, Saudi Arabia; mhagar@taibahu.edu.sa; 4 Department of Chemistry, Faculty of Science, Alexandria University, Alexandria 21321, Egypt

**Keywords:** azo chromene dyes, biological assessment, molecular docking, DFT calculations, SAR

## Abstract

The high biological activity of the chromene compounds coupled with the intriguing optical features of azo chromophores prompted our desire to construct novel derivatives of chromene incorporating azo moieties 4a-l, which have been prepared via a three-component reaction of 1-naphthalenol-4-[(4-ethoxyphenyl) azo], 1, with the benzaldehyde derivatives and malononitrile. The structural identities of the azo-chromene 4a-l were confirmed on the basis of their spectral data and elemental analysis, and a UV–visible study was performed in a Dimethylformamide (DMF) solution for these molecules. Additionally, the antimicrobial activity was investigated against four human pathogens (Gram-positive and Gram-negative bacteria) and four fungi, employing an agar well diffusion method, with their minimum inhibitory concentrations being reported. Molecules 4a, 4g, and 4h were discovered to be more efficacious against *Syncephalastrum racemosum* (RCMB 05922) in comparison to the reference drugs, while compounds 4b and 4h demonstrated the highest inhibitory activity against *Escherichia coli* (*E. coli*) in evaluation against the reference drugs. Moreover, their cytotoxicity was assessed against three different human cell lines, including human colon carcinoma (HCT-116), human hepatocellular carcinoma (HepG-2), and human breast adenocarcinoma (MCF-7) with a selection of molecules illustrating potency against the HCT-116 and MCF-7 cell lines. Furthermore, the molecular modeling results depicted the binding interactions of the synthesized compounds 3b and 3h in the active site of the *E. coli* DNA gyrase B enzyme with a clear SAR (structure–activity relationship) analysis. Lastly, the density functional theory’s (DFTs) theoretical calculations were performed to quantify the energy levels of the Frontier Molecular Orbitals (FMOs) and their energy gaps, dipole moments, and molecular electrostatic potentials. These data were utilized in the chemical descriptor estimations to confirm the biological activity.

## 1. Introduction

The last century has seen an exponential increase in the growth of cancerous masses and this has emerged as one of the primary global causes of mortality in this age. Due to the presence of genetic and environmental factors such as mutations, carcinogens, detrimental nutrition, and emissions, cancer incidence has been on the rise within both developing and developed countries [1]. To combat this, an entire cancer therapeutics industry has been established throughout the years, which is devoted to the research and discovery of novel cancer treatments, which include surgery, radiotherapy, chemotherapy, hormonal therapy, targeted drug therapy, and clinical trials [2]. However, complications arise with several of these methodologies, which introduce a necessity to enhance the performance of this therapeutics and drive the present search and discovery of better-performing antitumor agents.

A suitable candidate of these criteria is the family of chromene molecules, which have captivated cancer researchers for their diversified and assorted medicinal applications, specifically anti-inflammatory, anti-cancer, and anti-fungal [3,4,5,6,7,8,9]. The design and introduction of chromene compounds as innovative medicinal agents has been a central focus in the emerging drug industry. Venugopala and co-workers reported several chromene molecules performed a focal role in the treatment of various cancers either as natural or synthetic compounds [10]. Mccarroll and co-workers also reported that tephrosin, a viable anti-lung carcinoma drug, as well as Acronycine, a drug targeting colon, lung, and ovary tumors, obstruct the cell proliferation through their binding to the β-tubulin specific site, which subsequently induces apoptosis through microtubule polymerization [11]. Furthermore, chromene molecules are deemed ‘privileged medicinal scaffolds’ due to their unique pharmacological and biological activities [5,11,12,13,14,15,16,17,18,19,20,21,22,23,24,25,26].

The pharmacological and biochemical properties of 4*H*-chromenes inspire many researchers to synthesize new compounds featuring different aromatic rings fused to the chromene moiety and obtain more active derivatives, as they are reliant upon the pattern of substitution. Of these aromatic derivatives, azo chromophores have been utilized due to their comprehensive applications in analytical chemistry [27], pharmaceuticals [28], food industries [29], and optics [30,31,32,33]. Additionally, they have miscellaneous biological activities, such as antibacterial, antifungal, and anti-HIV properties [34]. The incorporation of a suitable heterocyclic moiety such as chromene derivatives with an azo linkage enhances their medicinal performance for particular usages, as antibacterial [34,35], antimicrobial [36,37,38], antioxidant [39], and other useful chemotherapeutic agents, and institute a fresh variety of chemical, physical, and biological applications [40]. Our work has been centered on the synthesis of novel chromene and chromene-based azo chromphores and the exploration of their biological performances. As an extension of our aforementioned work [41,42,43], herein, we present the synthesis and characterization of the novel azo-chromene molecules and investigate their in vitro antimicrobial activity and cytotoxic effects. Moreover, this work incorporates the calculation of theoretical parameters by the density functional theory (DFT) for the prepared molecules, which illustrate the optical/biological results in terms of the structural activity relationships (SAR) of the reported compounds [44,45,46].

## 2. Results and Discussion

### 2.1. Synthesis and Characterization

**Scheme 1:**
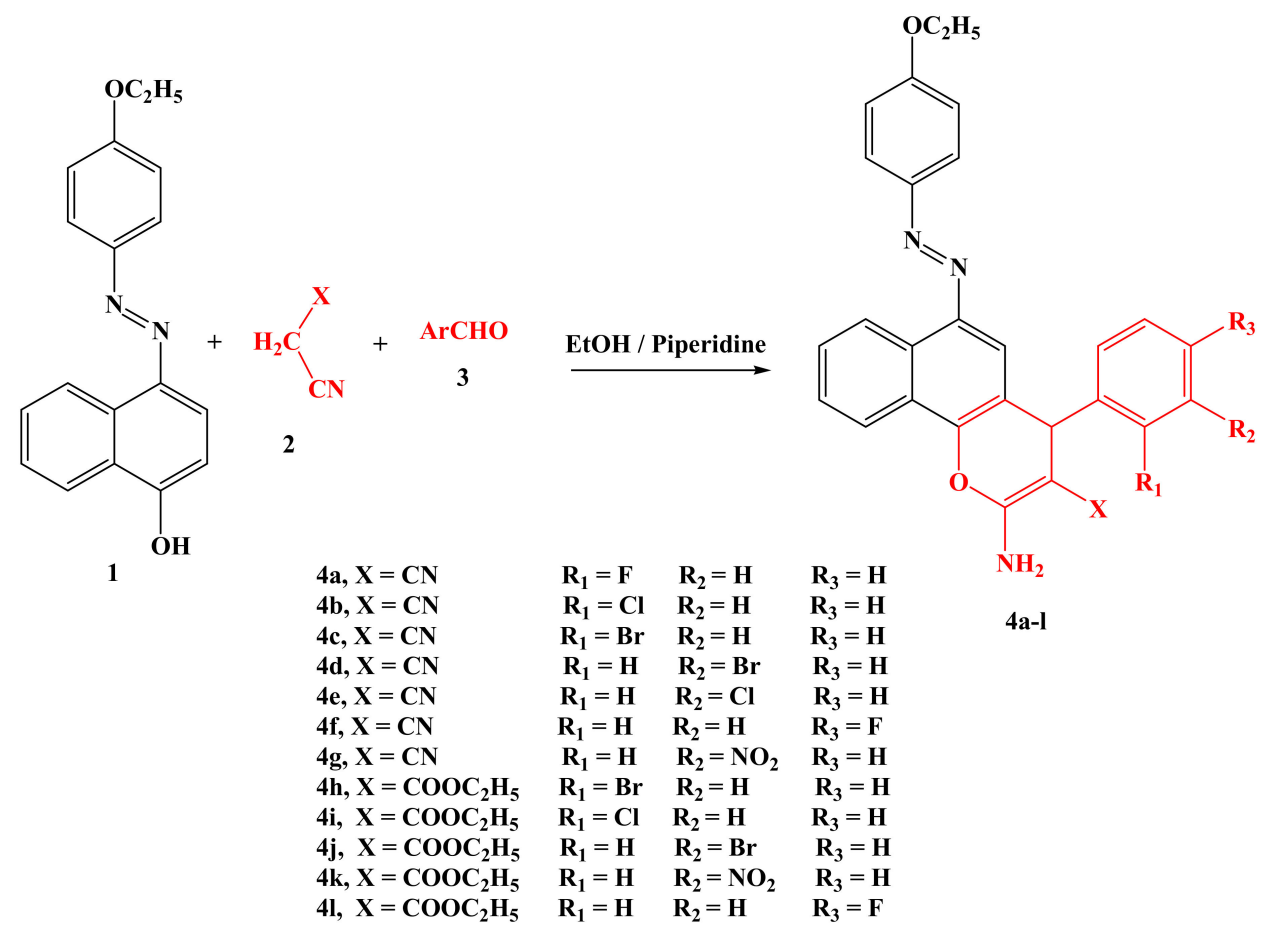
Synthesis of 2-amino-6-(4-ethoxyphenylazo)-4(-phenyl)-4*H*-benzo[*h*]chromene derivatives **4a-l.**

In our attempts to synthesize chromene derivatives, the three-component condensation reaction of 1-naphthalenol-4-[(4-ethoxyphenyl) azo] **1** with malononitrile and/or ethyl cyanoacetate **2** and the benzaldehyde containing either electron-donating (EDG) or -withdrawing (EWG) derivatives **3a–l** was executed in an ethanolic reflux solution using piperidine and afforded 2-amino-6-(4-ethoxyphenylazo)-4(-phenyl)-4*H*-benzo[*h*]chromene-3-carbonitrile derivatives in good yield, as illustrated in Scheme 1.

**Scheme 2:**
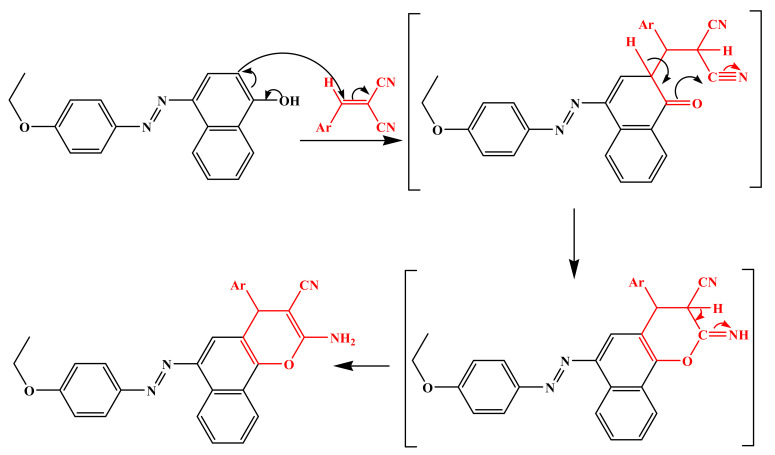
Mechanistic pathway of synthesis of chromene molecules **4a-l.**

The aforementioned Knoevenagal condensation, followed by the Michael addition adducts, is the fundamental methodology for this class of synthesis, and the plausible mechanism [47] is represented in Scheme 2.

The structures of these chromene compounds **4a-l** were confirmed with the aid of spectroscopic data. Case in point, the FT-IR spectroscopy of the acquired compounds revealed characteristic absorption bands between 2187 and 2210 for the CN groups while the NH_2_ stretches were in the range of 3336–3470 cm^−1^. Additionally, the ^1^H NMR spectra of the chromene compounds **4a-l** were obtained in DMSO-d_6_. As anticipated, the methyl protons appeared as a triplet set at 1.34–1.37 ppm with the H-4 pyran displaying signals at 5.07–5.49 ppm and the amine protons resonating at 6.71–7.11 ppm. Meanwhile, the singlet at 4.11–4.19 ppm corresponds to the methylene group. The rest of the aromatic protons resonate further downfield in the range of 6.62–8.97 ppm. The ^13^C NMR spectra of the synthesized molecules revealed the carbon atoms’ attachment to the methyl protons, resonating at 15.40–14.48 ppm, while the methylene carbons appeared at 64.58–64.14 ppm. In addition, the signal at 41.71–31.52 ppm corresponded to the C-4 of the pyran ring, and the quaternary carbon C-2, which is attached to the amine group, appeared in the range of 58.49–55.67 ppm. Lastly, the CN carbon resonated further downfield at 118.92–117.69 ppm, and the aromatic CH carbons exhibited signals between 144.06 and 111.75 ppm.

### 2.2. UV–Visible and Halochromism Studies

**Figure 1:**
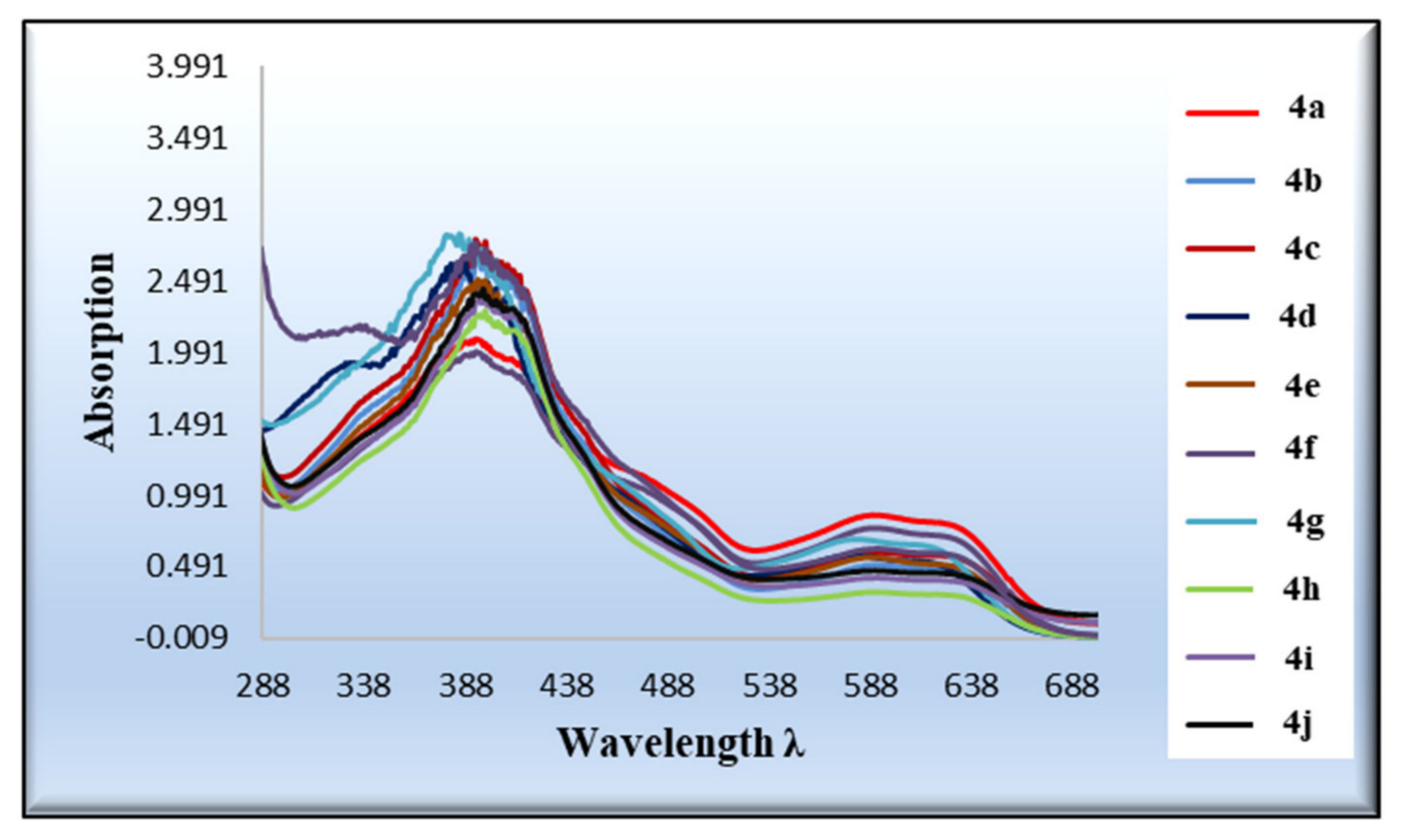
UV–vis absorption spectra of compounds **4a-j** (10 µM) in dimethylformamide (DMF).

The new highly colored molecules **4(a-j)** were dissolved in a Dimethylformamide (DMF) solution with a concentration of 10^−3^ M in order to examine their UV–Vis absorption spectroscopy. The spectra of all examined compounds demonstrated two comparable peaks around 393 and 591 nm. The first band appeared due to the n-π* transition with a substantial charge-transfer feature expressed in the peak’s broadness. Meanwhile, the longer wavelength band is attributed to the weak forbidden n-π*, the π-π*, and the solvated complex development via an intermolecular H-bonding between the chromene dyes and the DMF, Figure 1.

**Figure 2:**
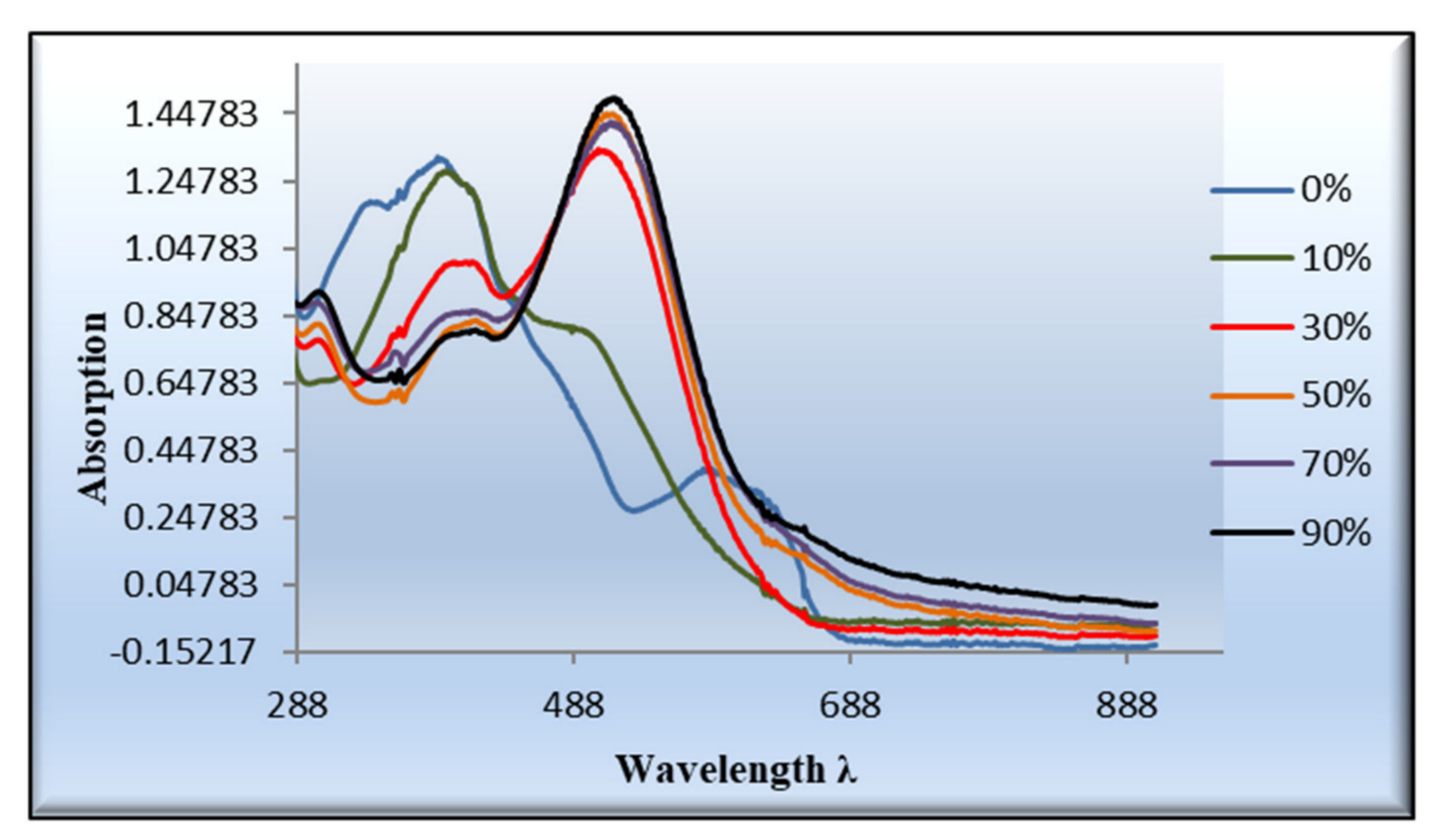
Wavelength of **4a** in the presence of increasing [H^+^] in a DMF.

**Figure 3:**
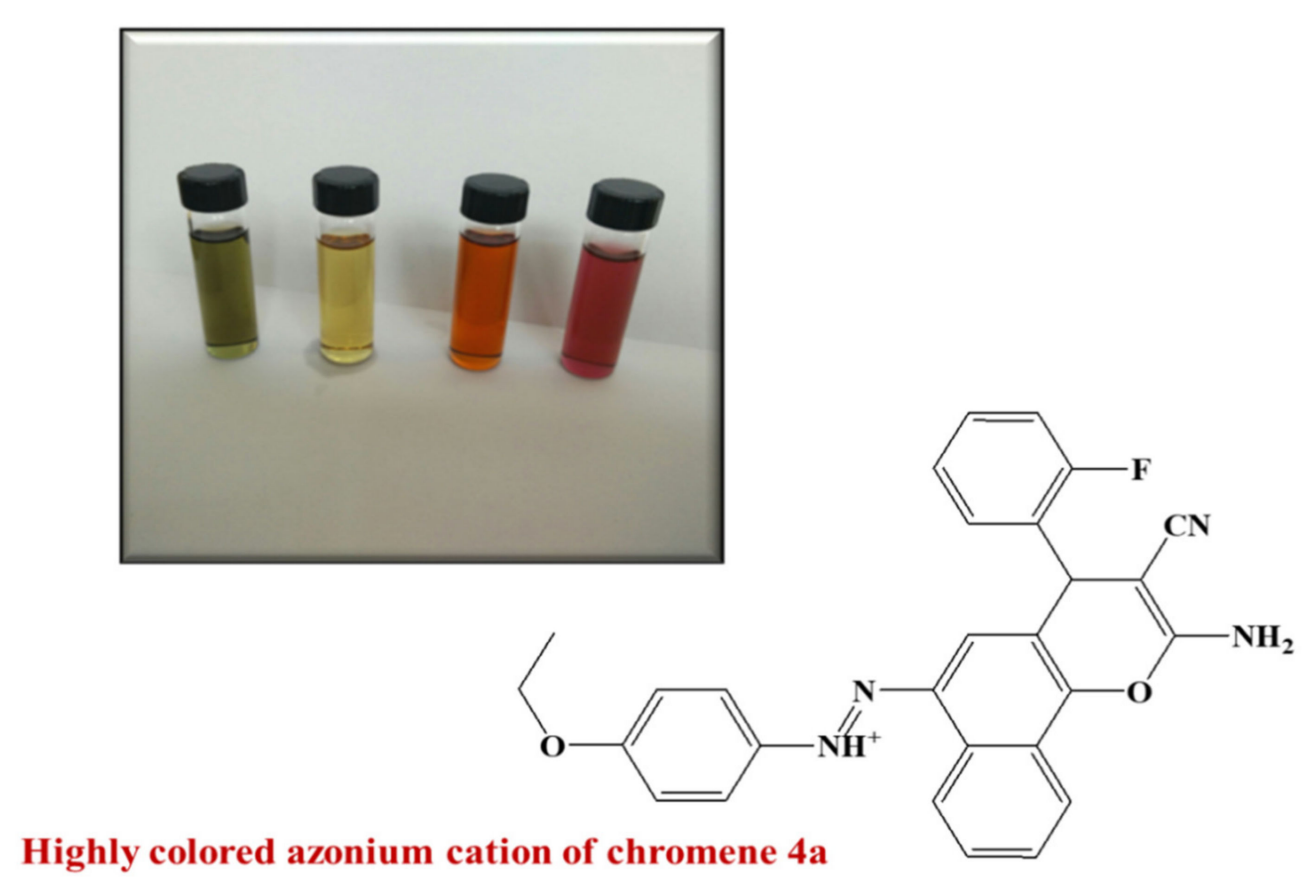
Color change of **4a** in the presence of increasing [H^+^] in a DMF.

The electronic absorption spectra of the azo chromophores are manipulated by the pH value, which influences the intensity and position of the wavelength maxima. The addition of an acid to the DMF solution of the azo chromene **4a-j** exerted a bathochromic shift in their absorption maxima, owing to their protonation and the establishment of the azonium configuration. Figure 2 illustrates the occurrence of the bathochromic shift of compound **4a** upon the addition of an acid, evidenced by the appearance of two longer λ_max_ values at 524 and 408 nm [48]. This behavior could be visualized through the gradual fluctuation of the color of the examined solution while altering the pH value, Figure 3.

### 2.3. Calculated Frontier Molecular Orbitals (FMOs)

**Figure 4:**
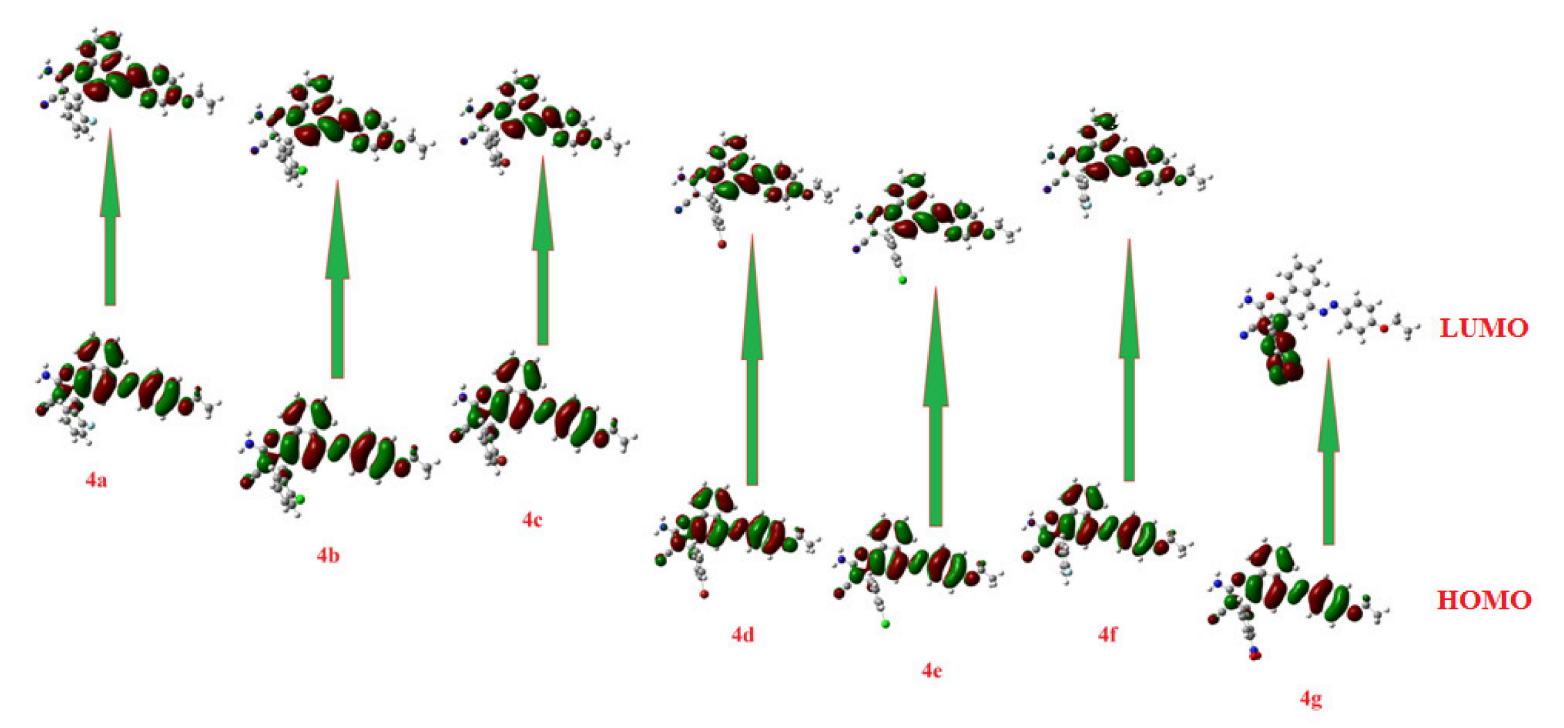
Molecular orbital spatial distribution and localization for the HOMO (highest occupied) and LUMO (lowest unoccupied) of **4a-g**.

**Figure 5:**
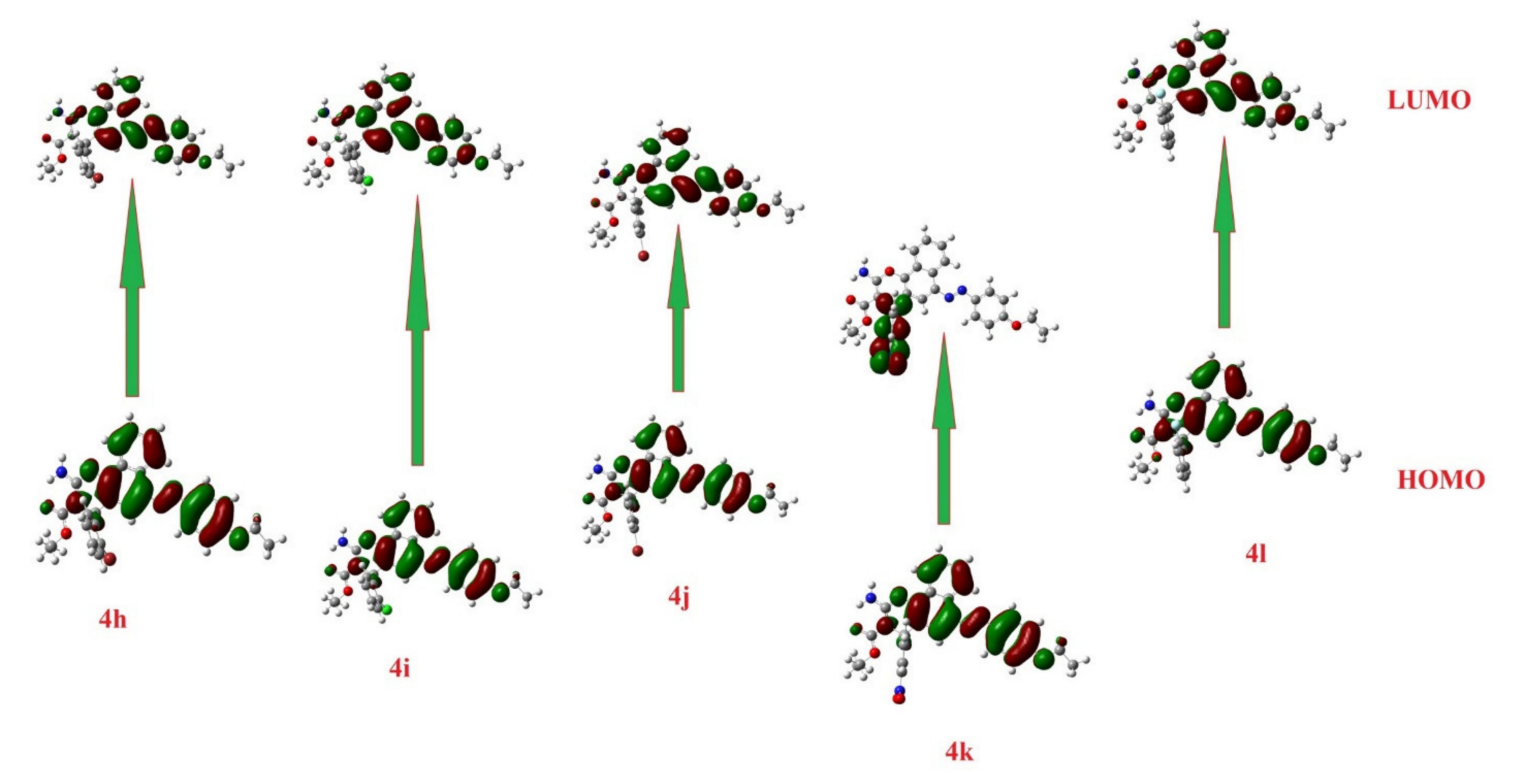
Molecular orbital spatial distribution and localization for the HOMO and LUMO of **4h-l**.

Table 1 and Figure 4 and Figure 5 demonstrate the isodensity projection of the calculated plots for the frontier molecular orbitals, both HOMO (highest occupied) and LUMO (lowest unoccupied), of all the prepared dyes **4a**-**l**. Additionally, they divulge their level of energy, energy gap, and maximum wavelength. It is notable that the energy levels of the FMOs and their energy gap are heavily affected by the electronic nature of the polar substituents and their position. These findings could be illustrated in terms of the degree of conjugation and the donating or the withdrawing ability of the polar group. Furthermore, a low bathochromic shift was observed by changing the attached halogen, which revealed the fluorine atom (**4a**) had less donation ability than the bromine atom (**4c**). However, the attachment of the halide atoms in the meta position (**4d** and **4e**) exhibited an extremely little bathochromic influence with respect to para one (**4f**), which could be explained in terms of the insignificant mesomeric effect of the halides atoms. On the other hand, the attachment of the nitro moiety (**4g**) displayed a substantial impact on the energy levels as well as the energy gap between the FMOs. For instance, the extra conjugation, resulting from the attachment of the nitro group, afforded a high bathochromic shift, while the high electron-withdrawing power of this moiety reduced the energy of the FMOs as well as their energy gap. Similar data were witnessed for the other series of the ethyl carboxylate derivatives (**4h-l**) with an equivalent impact on either the position or the type of the attached group.

### 2.4. Biological Screening

#### 2.4.1. Antimicrobial Screening

**Figure 6:**
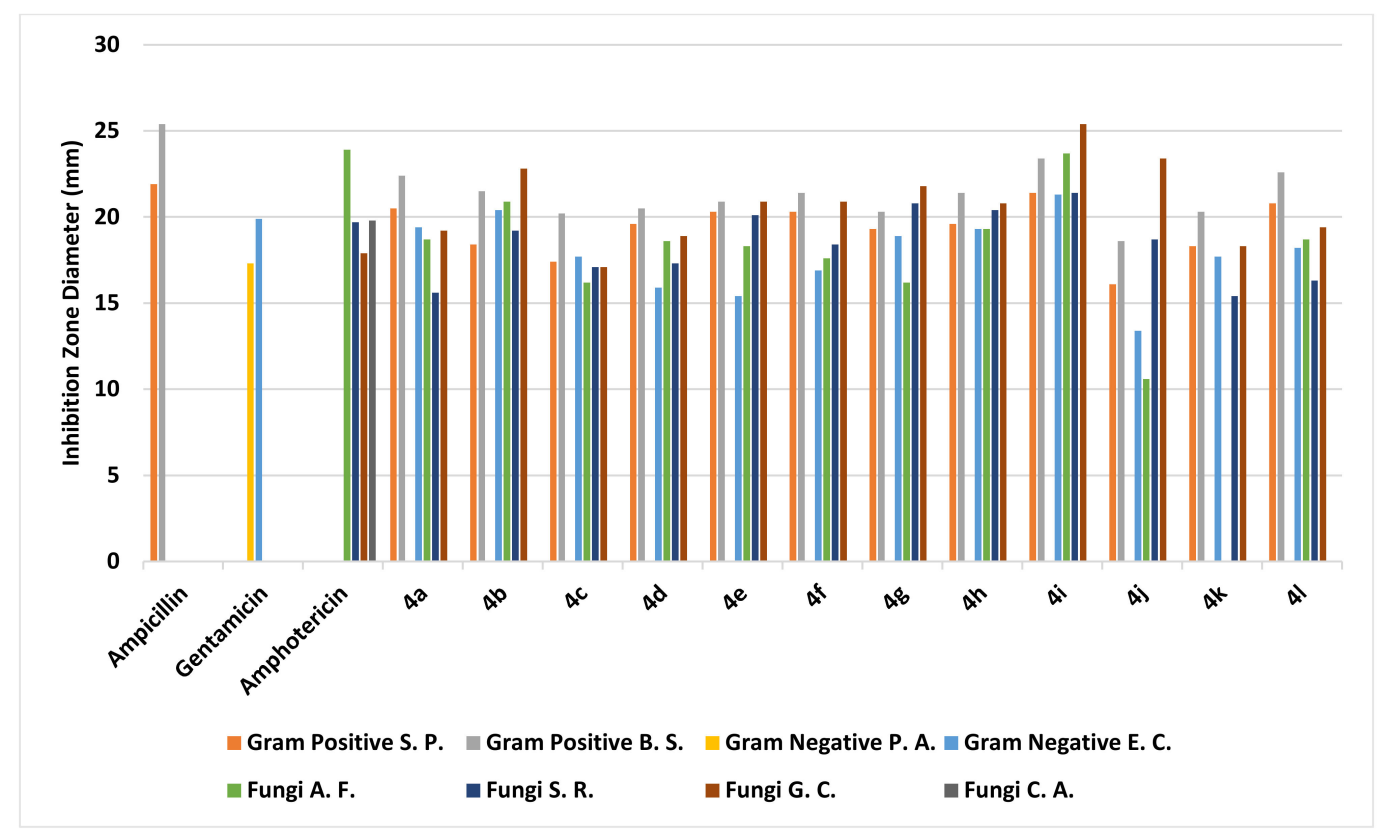
Evaluation of Inhibition zone values (IZ) of synthesized derivatives **4a-l**.

**Figure 7:**
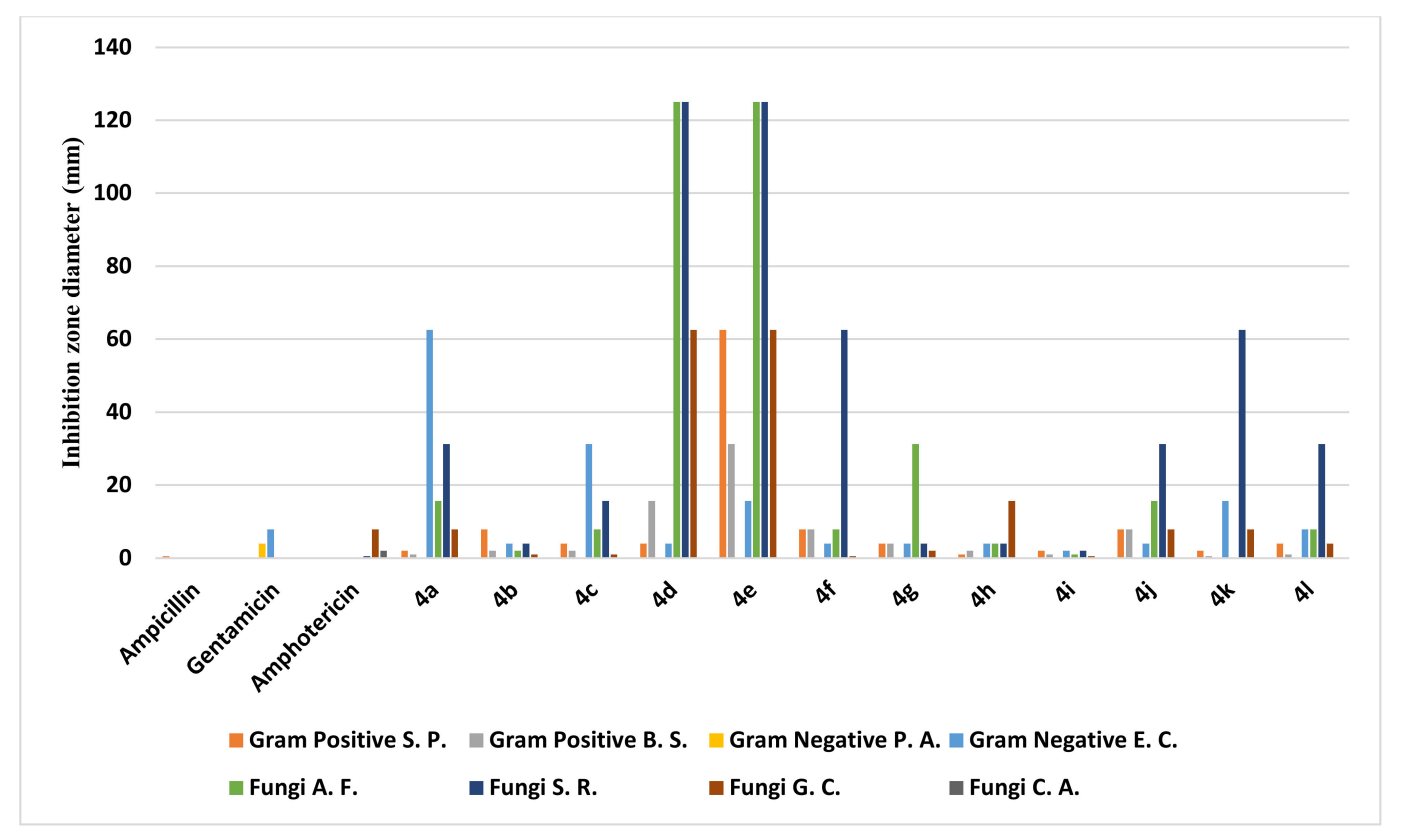
Evaluation of minimum inhibitory concentration values (MIC) of compounds **4a-l**.

The agar well diffusion method was used for the assessment of the antimicrobial and antifungal performance of the benzochromene derivatives 4a-l [49]. The inhibition zones and minimum inhibitory concentrations (MIC) were determined via a serial dilution method [49]. The bioassays involved two Gram-positive bacteria, two Gram-negative bacteria, and four fungi, as described in more detail in the Section 3. Ampicillin, Gentamicin, and Amphotericin B were employed as control drugs [50]. The observed inhibition zone (IZ) and minimum inhibitory concentrations (MIC) of the assessed molecules and the reference drugs are summarized in Table 2 and Table 3 and illustrated in Figure 6 and Figure 7. Among the synthesized chromophores, compounds **4a**, **4g**, **4h**, and **4i** were discovered to be more efficacious against *Syncephalastrum racemosum* (RCMB 05922) by an IZ range of 20.1–21.4 mm and a MIC value of 1.95 to 3.9 µg/mL in evaluation with the reference drugs. Meanwhile, the appraised molecules displayed potency against the *Aspergillus fumigatus* and *Geotrichum candidum* strains with IZ and MIC values comparable to the reference drugs. In the case of the Gram-negative bacteria, the highest inhibitory activity against *Escherichia coli* was demonstrated through an IZ range of 20.4 to 21.2 mm with a MIC = 3.9 µg/mL for derivatives **4b** and **4h** in comparison with the standard drugs. On the other hand, the synthesized compounds were slightly more active against *Streptococcus pneumoniae* and *Bacillus subtilis,* while all the investigated molecules did not exhibit any antimicrobial activity against *Pseudomonas aeruginosa* and *Candida albicans*. Generally, the investigation of the antimicrobial activity of the novel derivatives exhibited greater potency than the reference drugs against *E. coli* and *S. racemosum* and slight activity towards the Gram-positive bacteria *S. pneumoniae* and *B. subtilis*.

#### 2.4.2. Cytotoxic Screening

**Figure 8:**
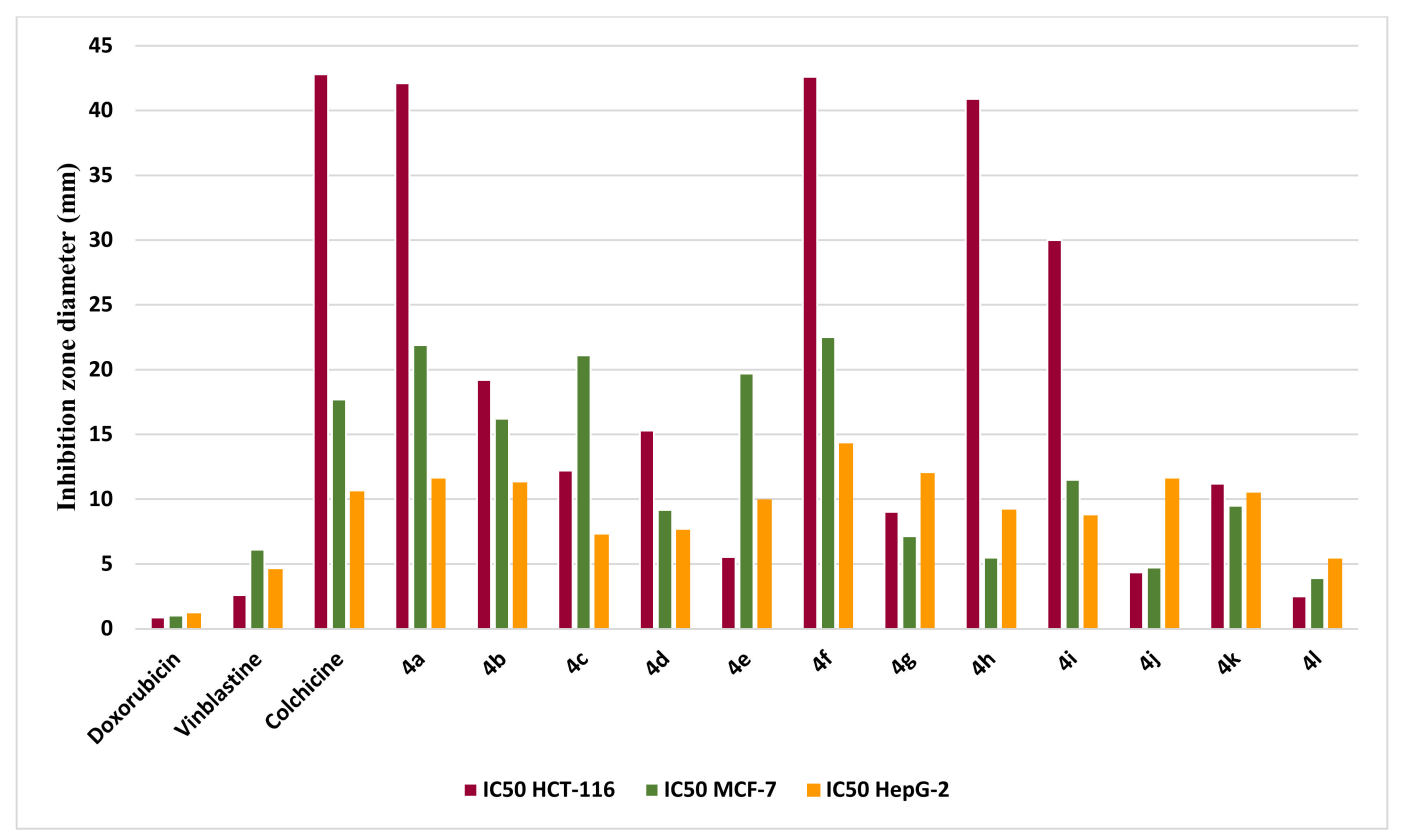
Evaluation of the cytotoxic activity of target compounds compared **4a**-**4l** to reference drug.

The in vitro cytotoxicity examination was performed by the MTT assay [51,52] against three human carcinoma cell lines: Human colon carcinoma (HCT-116), human breast adenocarcinoma (MCF-7), and human hepatocellular carcinoma (HepG-2). Doxorubicin, Vinblastine, and Colchicine were utilized as positive controls for this study. The inhibitory effects of the desired compounds, **4a-j**, on the growth of the three cell lines are illustrated in Table 4 and Figure 8. All compounds demonstrated comparable or slightly cytotoxic behavior in comparison to the reference drug. Additionally, the newly synthesized derivatives **4e**, **4h**, and **4j** exhibited good IC_50_ ranging (from 4.35 to 5.54 µg/mL) against the HCT-116 and MCF-7 cell lines while all the appraised molecules displayed less activity in case of the HepG-2 cell line.

### 2.5. Computational Studies and SAR Analysis

#### 2.5.1. Docking Studies

**Figure 9:**
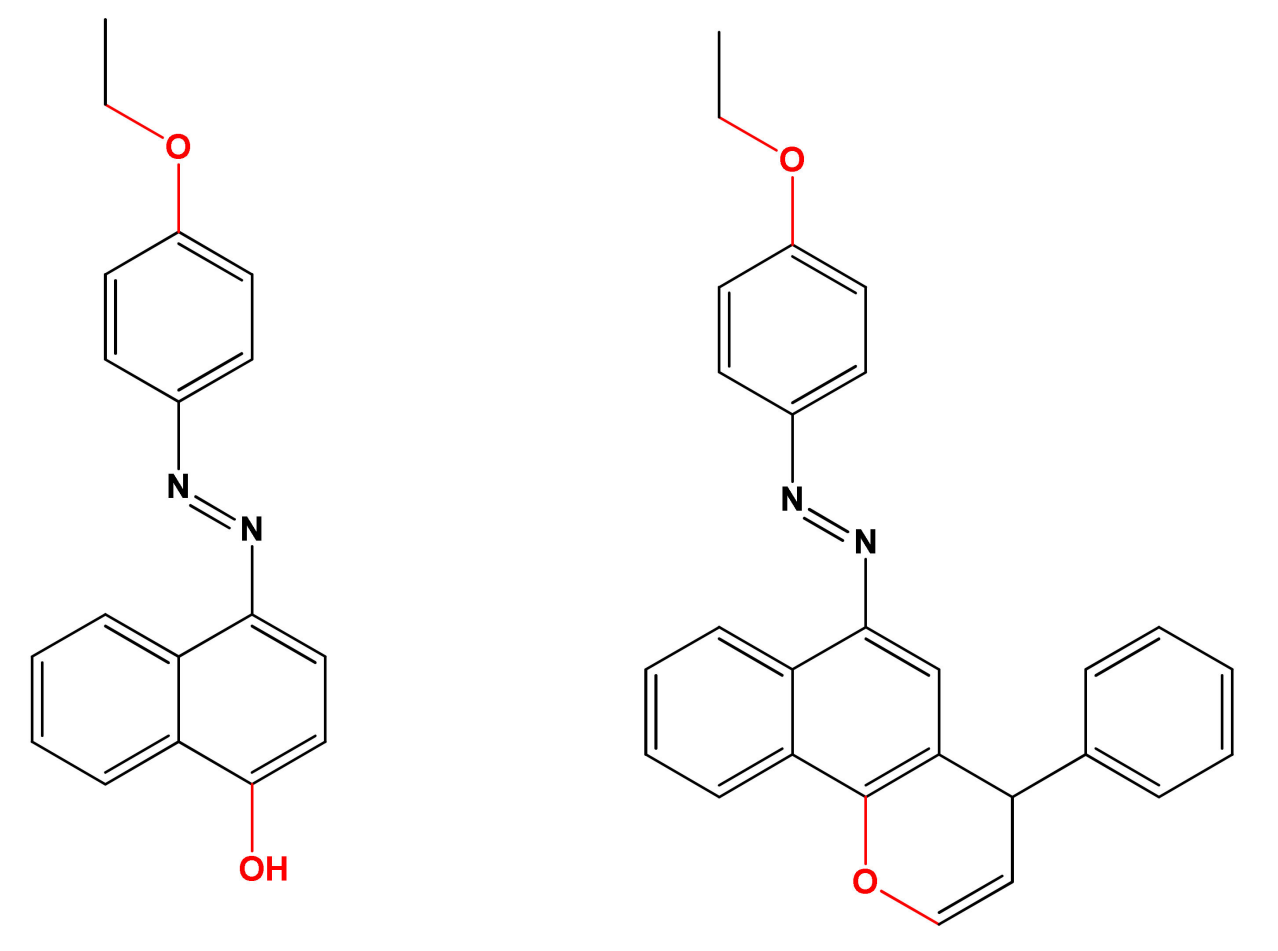
Structures of Fat Brown B and 1-(4-ethoxyphenyl)-2-(4-phenyl-4H-benzo[*h*]chromen-6-yl)diazene [55].

**Figure 10:**
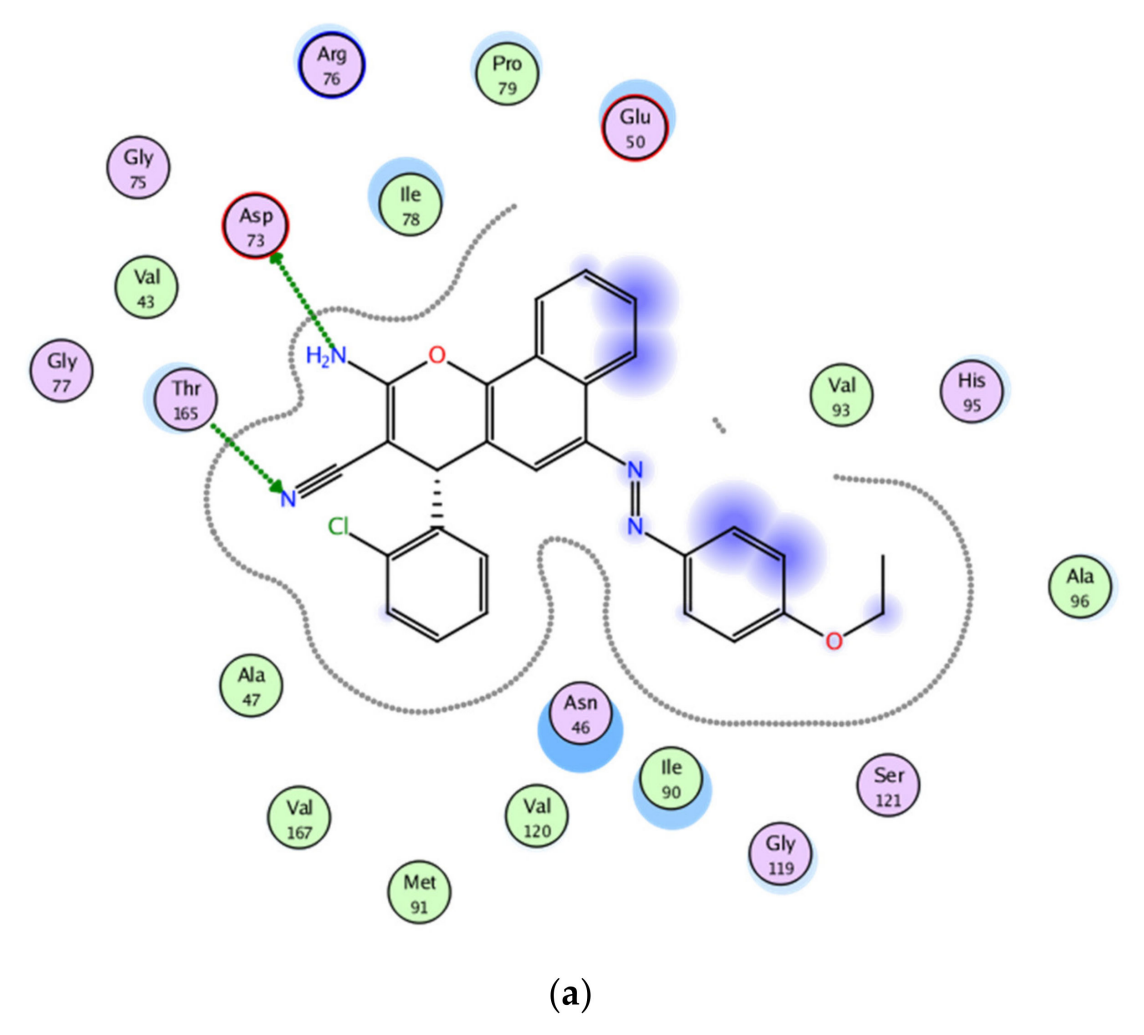

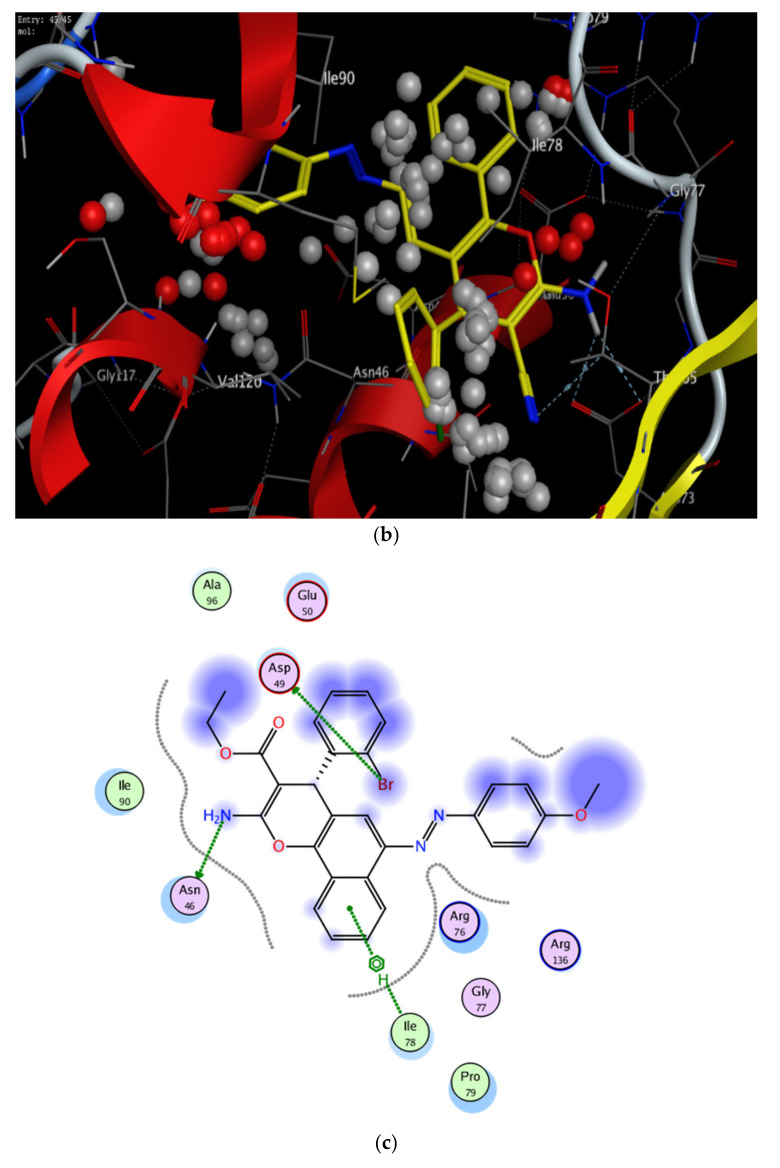

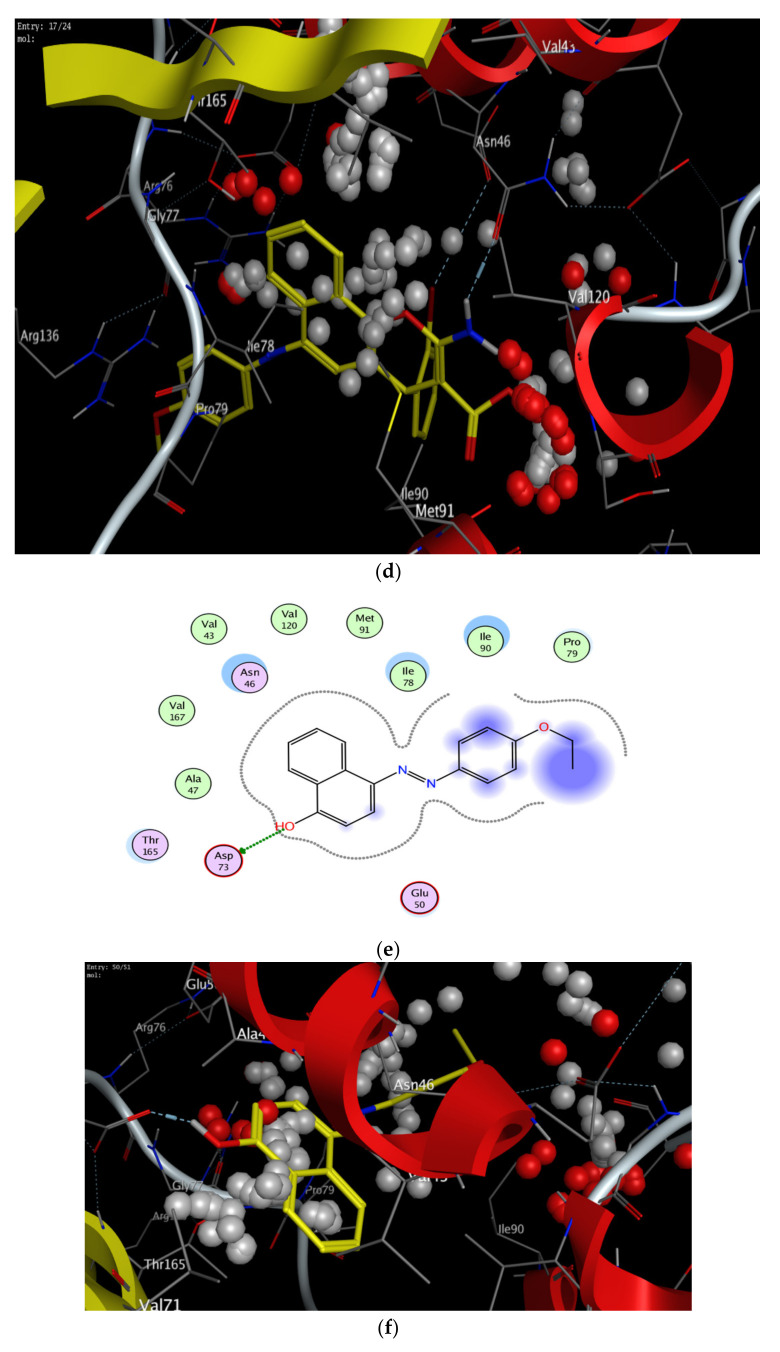

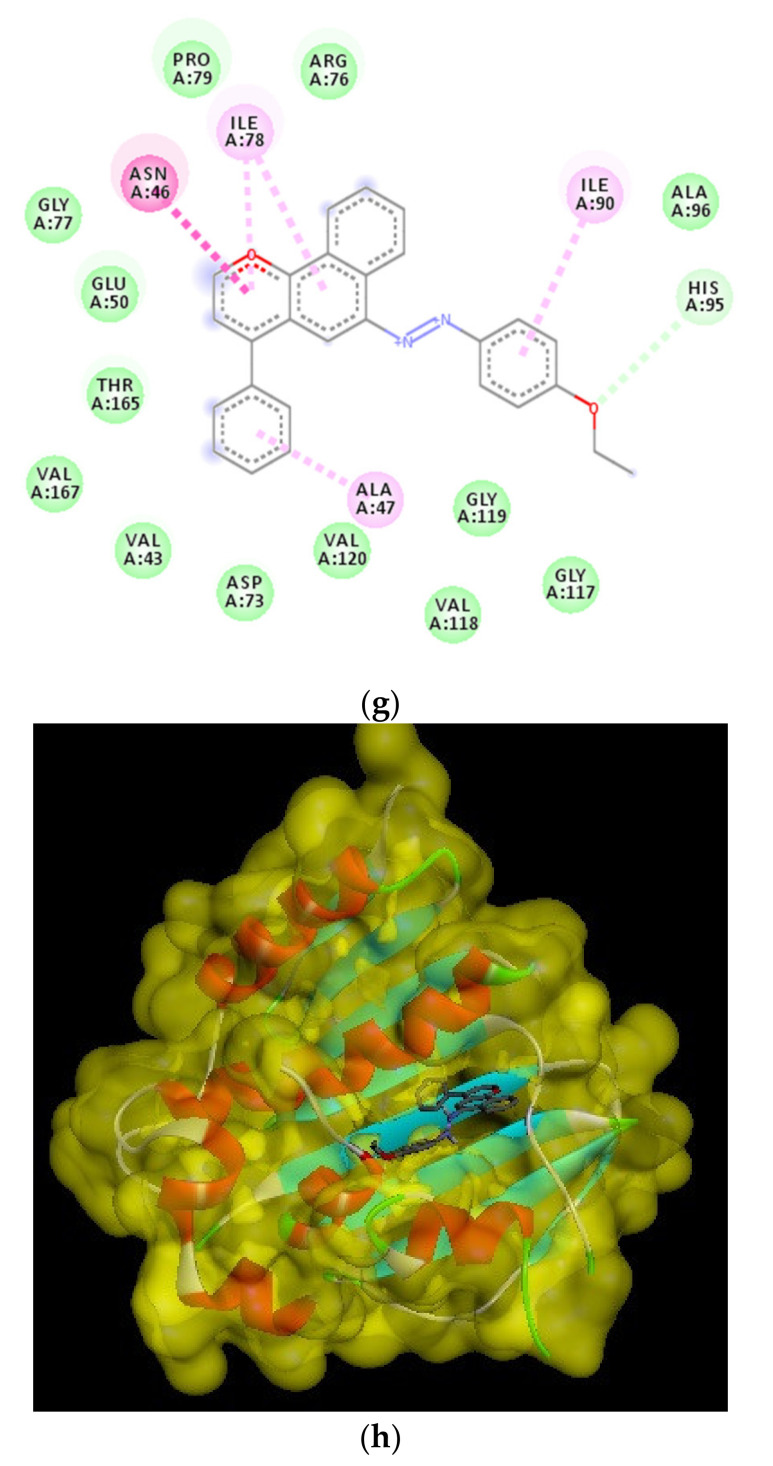
2D and 3D binding interaction of 1-(4-ethoxyphenyl)-2-(4-phenyl-4*H*-benzo[*h*]chromen-6-yl)diazene derivatives inside the enzyme. (**a**,**b**) Derivative **4b**, (**c**,**d**) derivative **4h**, (**e**,**f**) Fat brown B dye, and (**g**,**h**) unsubstituted chromene compounds.

The molecular docking calculations of the synthesized derivatives **4b** and **4h** were performed to gain insight into the plausible mechanism of the antibacterial activity of the target compounds, utilizing the DNA gyrase B subunit as it has been reported as a good target for studying inhibitory activity against these bacteria [46]. Meanwhile, the *E. coli* topoisomerase II DNA gyrase B (responsible for the supercoiling activity of DNA in bacteria) (PDB code: 1KZN; resolution 2.30 Å) was used by the docking module implemented in MOE softwares [50,53]. To validate these docking results, re-docking was performed using PyRx, an AutoDock Vina option that is based on scoring functions [54]. The Fat Brown B (compound 1, starting material) and 1-(4-ethoxyphenyl)-2-(4-phenyl-4H-benzo[*h*]chromen-6-yl)diazene (the hypothetical final product without any NH_2_, CN, COOC_2_H_5,_ and halogen group, (Figure 9) were utilized for comparing the binding affinity and the type of interactions of the synthesized compounds. Additionally, the least binding energy mode of the compounds has been studied. Figure 10 presents the best docking poses for the investigated chromene derivatives inside the topoisomerase II DNA gyrase B binding pocket. The 3D and 2D interaction of 1-(4-ethoxyphenyl)-2-(4-phenyl-4H-benzo[*h*]chromen-6-yl)diazene were visualized using BIOVIA Discovery studio visualizer.

The results of the docking experiments were summarized in Table 5. The 2D and 3D binding map of the target compounds **4b** and **4h** in the pocket is explained through three different fragments around the chromene scaffold: 8-NH_2_, 9-CN, and -Br, which belong to **4h**. These fragments formed stable hydrogen bonding interactions with a panel of corresponding pocket residues: Asp 73, Thr 165, Asn 46, Asp 49, and Ile 78 with different distances between 2.00 and 3.23 Å. Fat Brown B displayed diverse interactions, including a hydrogen bonding interaction with Asp 73 (2.45 Å), while no hydrogen bond was established with 1-(4-ethoxyphenyl)-2-(4-phenyl-4H-benzo[*h*]chromen-6-yl)diazene, and this might be the reason for their lower binding affinity with the target receptor. This proves the importance of the synthesized compounds bearing NH_2_, CN, COOC_2_H_5,_ and halogen group over the starting material, i.e., Fat Brown B and 1-(4-ethoxyphenyl)-2-(4-phenyl-4H-benzo[*h*]chromen-6-yl)diazene (the hypothetical final product without any NH_2_, CN, COOC_2_H_5_, and halogen group), Figure 9.

#### 2.5.2. Molecular Descriptors-Based SAR (Structure–Activity Relationship) Analysis

It is well documented that the energy values of the HOMOs and LUMOs for a specific molecule can be used as a qualitative tool to describe the molecule’s ability to donate or receive electrons from a receptor molecule [56,57,58]. The Frontier Molecular Orbital (FMO) levels and their corresponding energy gaps were measured to determine their antifungal [59,60], anticancer [61,62,63], antimicrobial [64,65,66,67], cytotoxic [68,69,70], and antiviral activities [56] as well as application in a new-drug-design field [71,72]. The FMOs also provide realistic qualitative evidence for the excitation characteristics for various chemical and pharmacological processes [56,73,74,75,76]. In addition, the type and degree of molecule-receptor binding interactions are defined by the energy level of the HOMOs and LUMOs, as well as their energy differences. Therefore, these values control the hydrogen bonding with the receptor, as well as nonbonding intermolecular interactions, such as hydrophobic effects.

Global hardness (η) could be used to predict a number of thermodynamic properties of the molecule including polar surface area, ovality, hydrophobicity, volume, electronegativity (χ), dipole moment (µ), and the level of charge transfer prevention of the molecule.

Furthermore, the electrophilicity (*ω*) could be calculated from the values of the electronegativity and chemical hardness and were also calculated from the energy levels of the FMOs, as seen in Table 6. The DFT analysis of the energy levels of the FMOs along with the energy differences of the prepared compounds **4a-l** is shown in Figure 4 and Figure 5. The levels of the prepared compounds’ HOMOs are in the range of −5.9 to −5.6 eV with compound **4g,** exhibiting the lowest energy level and **4l** the highest-lying level. On the other hand, the level of the LUMOs fluctuated between −3.1 eV to −2.4 eV with **4g,** demonstrating the high-lying LUMO and compound **4l at** the low-lying level. Therefore, molecule **4g** is the most capable of accepting electrons from neighboring receptors, and compound **4l** is the most capable of donating electrons to neighboring acceptors. The energy gaps between the FMO levels of the prepared compounds were in the range of 2.9 to 3.3 eV. It is clear that molecules **4a** and **4g** in comparison with the other molecules possess the highest and the lowest energy difference, respectively, and this could illustrate their high binding affinity in terms of the molecular docking scores for the measured biological activity.

The introduction of electron-withdrawing groups to the target scaffold appeared to positively contribute to the antimicrobial activity, especially against Gram-negative bacteria, as seen with compounds **4b** and **4h**. Moreover, quantum calculations confirmed these findings. The density distributions of the HOMO and LUMO surfaces are highlighted in Figure 4 and Figure 5 between representative molecules with different activity profiles. For the active compounds studied in this work, their HOMO character displayed less density in assessment with the LUMO characters, while the symmetrical distribution of these orbitals is related to lower biological activity. The less active compounds may interact through the charge transfer mechanism with some targets before reaching the biological bacterial DNA gyrase enzyme and inhibiting their passage through bacterial cell membranes. Another factor that could impact and influence the degree of the intermolecular binding affinity of the studied compounds with the protein is the dipole moment [77,78,79]. The calculated dipole moments of the prepared compounds **4a-l** were in the range of 2.5–10.3 Debye with molecule **4g** exhibiting the highest and **4l** the lowest. In addition, compounds **4l** and **4g** possessed the lowest and highest basicity scores, χ = 3.99 and 4.47, respectively, which could also be an illustration of their expected biological activity, Table 2.

Alternatively, the calculated lipophilicity revealed the ability of the drug-like molecules to flux in lipophilic parts, which allows passage through several membranes of the biological organs and results in cytotoxic molecules. The variation in the calculated log P (compound **4h** disclosed the lowest score of 6.4 while **4k** and **4i** scored 7.7) between such proposed compounds suggested a preference of **4k** and **4i** rather than **4h** for enzyme inhibition. Furthermore, compounds **4h** and **4l** demonstrated the highest H-bonding either in donation or the polar surface area with respect to the other compounds, Table 7.

The SAR analysis of these factors of quantum mechanical calculations of all compounds could be combined with different extent to illustrate their biological activity by describing the degree of the binding affinity of the compounds with the active sites of the main protein. In addition, to validate the evidence about the reactivity of the compounds under investigation as enzyme inhibitors, the molecular electrostatic potential (MEP) is an important parameter to be predicted since the MEP is a sign about the molecular size and shape of the positive, negative and the neutral electrostatic potential, and can be a tool for expecting physiochemical property relationships with the molecular structure. Furthermore, the molecular electrostatic potential is a useful tool in the prediction of the susceptibility of the studied compounds towards electrophiles and nucleophiles.

**Figure 11:**
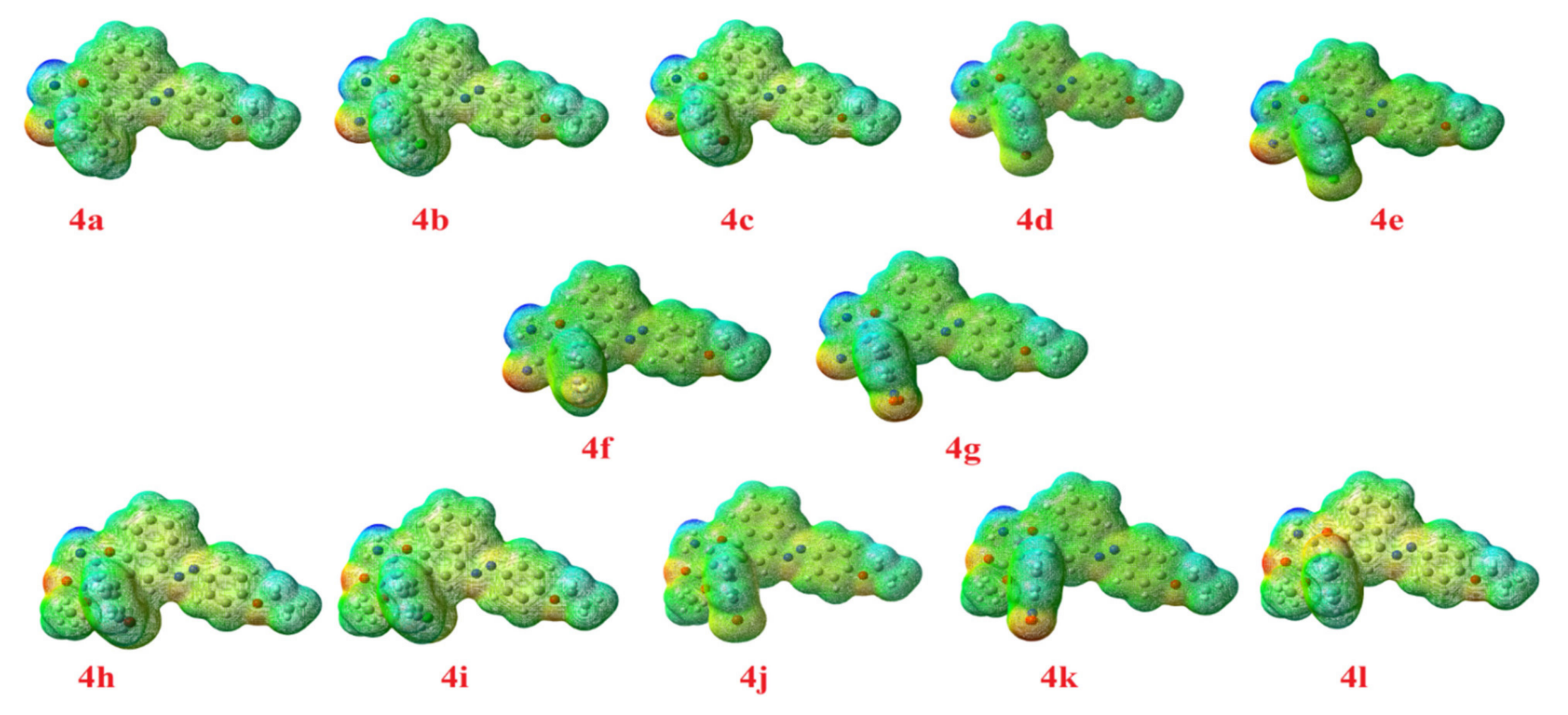
The molecular electrostatic potential for the prepared compounds (**4a-l**), blue color indicates hydrophobic regions and red color denotes hydrophilic polar areas.

The molecular electrostatic potential (MEP) was calculated by the same method under the same base sets as the previous calculations, Figure 11**.** In the MEP, the higher negative region is the favored site for electrophilic attack shown in the red color. So, an attacking electrophile will be attracted by the negatively charged sites and the opposite situation for the blue regions. It is obvious that the molecular size and the shape as well as the orientation of the negative, positive, and the neutral electrostatic potential are varied according to the electronic nature of the compound as well as the electronegativity of the attached groups. The difference in the mapping of the electrostatic potential around the compounds could be responsible for the extent of binding affinity of the studied compounds to the active site of the receptor.

## 3. Materials and Methods

### 3.1. Materials and Instrumentation

Chemicals and solvents were purchased from Sigma-Aldrich (Oakville, ON, Canada) and Alfa Aesar (Tewksbury, MA, USA) and were used as received. Compound **1** was purchased from Sigma-Aldrich (Oakville, ON, Canada). Melting points were determined in open capillaries using an electrothermal apparatus (Stuart Scientific, Stone, SFD, UK) and are uncorrected. The progress of the reactions was monitored using thin-layer chromatography (TLC) on Merck silica gel 60 F254 plates. Infrared (IR) spectra were recorded using Bruker Alpha FT-IR Spectrometer (Bruker, Billerica, MA, USA) as pressed KBr pellets. ^1^H-NMR and ^13^C-NMR spectra were recorded on 300 MHz and at 75 MHz, respectively, on a Bruker Avance Spectrometer (Bruker, Billerica, MA, USA) in DMSO-d_6_ with tetramethylsilane as an internal standard. The elemental analyses for C, H, and N were performed using an Exeter Analytical, Inc. CE-440 Elemental Analyser (Chelmsford, MA, USA). UV–vis absorption measurements were performed using a HP8543 UV–vis spectrophotometer.

### 3.2. Biological Studies

#### 3.2.1. Antimicrobial Screening

The microorganism inoculums were uniformly spread using sterile cotton swabs on a sterile Petri dish malt extract agar (for fungi) and nutrient agar (for bacteria). One hundred cubic millimeters of each sample were added to each well (10-mm-diameter holes were cut in the agar gel, 20 mm apart from one another). The systems were incubated for 24–48 h at 37 °C (for bacteria) and at 28 °C (for fungi). After incubation, microorganism growth was observed. Inhibition zones of the bacterial and fungal growth were measured in millimeters. Tests were performed in triplicate [80,81].

#### 3.2.2. Cytotoxic Screening

All cell lines including HCT-116 (human colon carcinoma), HepG-2 (human hepatocellular carcinoma), and MCF-7 (human breast adenocarcinoma) were received from the American Type Culture Collection (ATCC, Rockville, MD). The cells were grown as monolayers on RPMI-1640 medium (Lonza, Belgium) supplemented with 50 µg/mL gentamycin and 10% inactivated FCS (fetal calf serum). The potential cytotoxicity of the target compounds was evaluated using the method published by Gangadevi and Muthumary [82]. The cells adhered at the bottom of the wells in a 96-well microliter plate incubated for 24 h at 37 °C in a humidified incubator with 5% CO_2_ and then washed with sterile PBS (phosphate-buffered saline, 10 mM pH 7.2). The cells were then treated with 100 µL from different dilutions of tested sample in a fresh maintenance medium and incubated at 37 °C. Negative control samples were left untreated and positive controls containing doxorubicin were also evaluated for comparison as a reference drug. For each concentration of each test sample, six wells were used and observation was conducted every 24 h using an inverted microscope. The surviving cells were stained with crystal violet followed by cell lysing using 33% glacial acetic acid and then mixed. The absorbance at 590 nm using a microplate reader (SunRise, TECAN, Inc, USA) was measured and the absorbance values from untreated cells were considered as 100% proliferation [51,83]. Using a microplate reader, the number of viable cells was determined and the percentage of viability was calculated as [1−(ODt/ODc)]× 100%, where ODt is the mean optical density of wells treated with the tested sample and ODc is the mean optical density of untreated cells. A plot of the number of viable cells against the drug concentration yielded the survival curve of each tumor cell line after treatment with the specified compound and then the 50% inhibitory concentration (IC_50_) was estimated from the graph.

### 3.3. Molecular Modeling

The newly synthesized compounds were docked into the crystal structure of *E. coli* topoisomerase II DNA gyrase B (PDB code 1KZN). The MOE software [84] was used for all docking calculations. The MOE tools package was employed to generate the docking input files and to analyze the docking results. All non-polar hydrogens, clorobiocin, and crystal water molecules were removed prior to the calculations, and the protonation of the enzyme was carried out and was energy minimized. In each case, 100 docked structures were generated using genetic algorithm searches. The 3D structures of newly synthesized compounds were drawn in MOE, and the protonation of ligands was carried out. The energy of compounds was minimized up to 0.05 gradient using GBVI/WSA dG force field. These data were saved in the database as input file MOE. Root-mean-square deviation (RMSD) values were calculated, and initial ligand binding modes were plotted. Protein–ligand interaction plots were generated using MOE 2012.10. Quantum mechanical calculations and surface molecular orbitals were generated by a simulation module in MOE software.

### 3.4. Computational Methods and Calculations

The theoretical calculations for the investigated compounds were carried out by Gaussian 09 software (Vicenza, Italy). DFT/B3LYP methods using 6-31G (d, p) basis set was selected for the calculations. The geometries were optimized by minimizing the energies with respect to all geometrical parameters without imposing any molecular symmetry constraints. The structures of the optimized geometries had been drawn with Gauss View. Moreover, the calculated frequencies were carried out using the same level of theory. The frequency calculations showed that all structures were stationary points in the geometry optimization method with none imaginary frequency.

### 3.5. Synthesis

#### 3.5.1. General Procedure for the Synthesis of 2-Amino-6-(4-Ethoxyphenylazo)-4(-Phenyl)-4H-Benzo[*h*]Chromene Derivatives

A mixture of compound 1 (2.3 mmol), malononitrile or ethyl cyanoacetate (2.3 mmol), and aryl aldehyde (2.3 mmol) in ethanol (5 mL) with a few drops of pipredine was added. The reaction mixture was stirred at reflux. After completion of the reaction (monitored by TLC), the mixture was cooled and filtered, washed with ethanol and hexane to afford **4a-l**.

#### 3.5.2. 2-Amino-6-(4-Ethoxyphenylazo)-4-(2-Fluoro-Phenyl)-4H-Benzo[*h*]Chromene-3-Carbonitrile (4a)

Brown solid (85%), m.p. 205 °C; IR (KBr) cm^−1^: 3469, 3280 (NH_2_), 2925, 2891 (CH), 2201 (CN), 1571 (N = N); ^1^H NMR (300 MHz, DMSO) δ 8.87–8.83 (m, 1H, Ar-H), 8.39–8.34 (m, 1H, Ar-H), 7.94 (d, *J* = 8.9 Hz, 2H, Ar-H), 7.81–7.77 (m, 2H, Ar-H), 7.41 (s, 1H, Ar-H), 7.37–7.28 (m, 4H, Ar-H, NH_2_), 7.22–7.14 (m, 2H, Ar-H), 7.10 (d, *J* = 9.0 Hz, 2H, Ar-H), 5.27 (s, 1H, H4), 4.14 (q, *J* = 6.7 Hz, 2H, CH_2_), 1.36 (t, *J* = 6.8 Hz, 3H, CH_3_); ^13^C NMR (75 MHz, DMSO) δ 162.30 (C-2), 160.96, 147.53, 146.07, 143.96, 132.72 (Ar-C), 132.55, 131.35 (Ar-CH), 131.15 (Ar-C), 130.35, 128.81 (Ar-CH), 128.32 (Ar-C), 125.76, 124.11, 121.98, 120.85 (Ar-CH), 117.80 (CN), 116.95 (Ar-C), 116.67, 115.90, 112.18 (Ar-CH), 64.57 (CH_2_), 55.67 (C-3), 31.52 (CH-4), 15.39 (CH_3_); C_28_H_21_N_4_O_2_F; Calculated: C, 72.40; H, 4.56; N, 12.06. Found: C, 73.05; H, 4.50; N, 11.87.

#### 3.5.3. 2-Amino-6-(4-Ethoxyphenylazo)-4-(2-Chloro-Phenyl)-4H-Benzo[*h*]Chromene-3-Carbonitrile (4b)

Greenish-brown solid (87%), m.p. 219 °C; IR (KBr) cm^−1^: 3466, 3326 (NH_2_), 2995, 2973, 2932 (CH), 2197 (CN), 1567 (N = N); ^1^H NMR (300 MHz, DMSO) δ 8.81-8.79 (m, 1H, Ar-H), 8.37-8.34 (m, 1H, Ar-H), 7.90 (d, *J* = 8.8 Hz, 2H, Ar-H), 7.78–7.75 (m, 2H, Ar-H), 7.45 (d, *J* = 7.1 Hz, 1H, H), 7.40-7.21 (m, 6H, Ar-H, NH_2_), 7.07 (d, *J* = 8.8 Hz, 2H, Ar-H), 5.46 (s, 1H, H4), 4.11 (q, *J* = 6.7 Hz, 2H, CH_2_), 1.35 (t, *J* = 6.8 Hz, 3H, CH_3_); ^13^C NMR (75 MHz, DMSO) δ 162.32 (C-2), 160.86, 147.49, 146.03, 143.92, 142.72 (Ar-C), 133.01, 132.22, 131.38 (Ar-CH), 130.93 (Ar-C), 129.98, 128.87, 128.35 (Ar-CH), 125.78 (Ar-C), 124.07, 123.97, 122.03, 120.71 (Ar-CH), 117.69 (CN), 115.91 (Ar-CH), 111.87 (Ar-C), 64.57 (CH_2_), 55.78 (C-3), 31.53 (CH-4) 15.39 (CH_3_); C_28_H_21_N_4_O_2_Cl; Calculated: C, 69.92; H, 4.40; N, 11.65. Found: C, 71.35; H, 4.02; N, 11.75.

#### 3.5.4. 2-Amino-6-(4-Ethoxyphenylazo)-4-(2-Bromo-Phenyl)-4H-Benzo[*h*]Chromene-3-Carbonitrile (4c)

Brown solid (83%), m.p. 208 °C; IR (KBr) cm^−1^: 3468, 3326 (NH_2_), 2995, 2973, 2932 (CH), 2198 (CN), 1569 (N = N); ^1^H NMR (300 MHz, DMSO) δ 8.86–8.80 (m, 1H, Ar-H), 8.39–8.34 (m, 1H, Ar-H), 7.92 (d, *J* = 9.0 Hz, 2H, Ar-H), 7.80–7.76 (m, 2H, Ar-H), 7.64 (d, *J* = 7.5 Hz, 1H, Ar-H), 7.40–7.27 (m, 5H, Ar-H, NH_2_), 7.25–7.19 (m, 1H, Ar-H), 7.09 (d, *J* = 9.0 Hz, 2H, Ar-H), 5.49 (s, 1H, H4), 4.13 (q, *J* = 6.9 Hz, 2H, CH_2_), 1.36 (t, *J* = 6.9 Hz, 3H, CH_3_); ^13^C NMR (75 MHz, DMSO) δ 162.32 (C-2), 160.78, 147.46, 145.89, 143.91 (Ar-C), 134.10, 132.45, 131.39, 130.24 (Ar-CH), 129.54 (Ar-C), 128.86, 128.34, 125.78 (Ar-CH), 124.11(Ar-C), 123.96 (Ar-CH), 123.32 (Ar-C), 122.06, 120.65 (Ar-CH), 117.80 (CN), 115.89 (Ar-CH), 111.75 (Ar-C), 64.56 (CH_2_), 56.01 (C-3), 31.53 (CH-4), 15.38 (CH_3_); C_28_H_21_N_4_O_2_Br; Calculated: C, 64.01; H, 4.03; N, 10.66. Found: C, 65.78; H, 3.67; N, 10.78.

#### 3.5.5. 2-Amino-6-(4-Ethoxyphenylazo)-4-(3-Bromo-Phenyl)-4H-Benzo[*h*]Chromene-3-Carbonitrile (4d)

Brown solid (86%), m.p. 217 °C; IR (KBr) cm^−1^: 3466, 3335 (NH_2_), 2975, 2935, 2883 (CH), 2193 (CN), 1566 (N = N); ^1^H NMR (300 MHz, DMSO) δ 8.89–8.80 (m, 1H, Ar-H), 8.37–8.36 (m, 1H, Ar-H), 7.95 (d, *J* = 9.0 Hz, 2H, Ar-H), 7.81-7.77 (m, 2H, Ar-H), 7.50 (s, 1H, Ar-H), 7.46-7.42 (m, 2H, Ar-H), 7.34–7.29 (m, 4H, Ar-H, NH_2_), 7.12 (d, *J* = 9.0 Hz, 2H, Ar-H), 5.09 (s, 1H, H4), 4.15 (q, *J* = 6.9 Hz, 2H, CH_2_), 1.37 (t, *J* = 6.9 Hz, 3H, CH_3_); ^13^C NMR (75 MHz, DMSO) δ162.33 (C-2), 160.68, 149.15, 147.55, 145.82 (Ar-C), 144.06, 132.04, 131.33 (Ar-CH), 131.20 (Ar-C), 130.94, 128.88, 128.36 (Ar-CH), 127.83 (Ar-C), 125.78, 124.22 (Ar-CH), 122.89 (Ar-C), 122.05, 120.89 (Ar-CH), 118.38 (CN), 115.93 (Ar-CH), 112.48 (Ar-C), 64.58 (CH_2_), 56.89 (C-3), 31.53 (CH-4), 15.40 (CH_3_); C_28_H_21_N_4_O_2_Br; Calculated: C, 64.01; H, 4.03; N, 10.66. Found: C, 66.27; H, 3.77; N, 10.87.

#### 3.5.6. 2-Amino-6-(4-Ethoxyphenylazo)-4-(3-Chloro-Phenyl)-4H-Benzo[*h*]Chromene-3-Carbonitrile (4e)

Dark brown solid (88%), m.p. 212 °C; IR (KBr) cm^−1^: 3470, 3336 (NH_2_), 2979, 2927, 2875 (CH), 2194 (CN), 1566 (N = N); ^1^H NMR (300 MHz, Acetone) δ 8.97-8.90 (m, 1H-Ar-H), 8.44-8.38 (m, 1H-Ar-H), 7.98 (d, *J* = 8.9 Hz, 2H, Ar-H), 7.80–7.73 (m, 2H, Ar-H), 7.55 (s, 1H, Ar-H), 7.43-7.27 (m, 4H, Ar-H, NH_2_), 7.15 (d, *J* = 11.7 Hz, 1H, Ar-H), 7.12 (d, *J* = 9.0 Hz, 2H, Ar-H), 6.62 (s, 1H, Ar-H), 5.11 (s, 1H, H4), 4.19 (q, *J* = 7.0 Hz, 2H, CH_2_), 1.43 (t, *J* = 7.0 Hz, 3H, CH_3_); ^13^C NMR (75 MHz, Acetone) δ 162.40 (C-2), 148.39, 147.68, 144.49, 134.54, 131.60 (Ar-C), 130.95, 128.32 (Ar-CH), 128.10 (Ar-C), 127.70, 127.05, 125.34 (Ar-CH), 125.16 (Ar-C), 124.34, 123.77, 122.49, 121.60, 119.23 (Ar-CH), 117.95 (CN), 115.28 (C-Ar-CH), 112.22 (Ar-C), 64.14 (CH_2_), 58.49 (C-3), 41.71 (CH-4), 14.48 (CH_3_); C_28_H_21_N_4_O_2_Cl; Calculated: C, 69.92; H, 4.40; N, 11.65. Found: C, 70.17; H, 4.21; N, 11.70.

#### 3.5.7. 2-Amino-6-(4-Ethoxyphenylazo)-4-(4-Fluoro-Phenyl)-4H-Benzo [*h*] Chromene-3-Carbonitrile (4f)

Greenish-brown solid (84%), m.p. 214 °C; IR (KBr) cm^−1^: 3466, 3278 (NH_2_), 2979, 2935, 2869 (CH), 2187 (CN), 1567 (N = N); ^1^H NMR (300 MHz, DMSO) δ 8.86–8.81 (m, 1H, Ar-H), 8.38–8.35 (m, 1H, Ar-H), 7.95 (d, *J* = 8.9 Hz, 2H, Ar-H), 7.81–7.76 (m, 2H, Ar-H), 7.41 (s, 1H, Ar-H), 7.35–7.27 (m, 4H, Ar-H, NH_2_), 7.19–7.09 (m, 4H, Ar-H), 5.07 (s, 1H, H4), 4.15 (d, *J* = 7.0 Hz, 2H, CH_2_), 1.37 (t, *J* = 6.9 Hz, 3H, CH_3_); ^13^C NMR (75 MHz, DMSO) δ 162.31(C-2), 160.51, 147.54, 145.74, 143.99, 142.69, 131.26 (Ar-C), 130.59, 130.48 (Ar-CH), 128.81 (Ar-C), 128.32, 125.76, 125.24 (Ar-CH), 124.21 (Ar-C), 123.98, 122.02 (Ar-CH), 120.99 (Ar-C), 118.92 (CN), 116.61, 116.32, 115.92, 112.60 (Ar-CH), 64.57 (CH_2_), 57.34 (C-3), 31.54 (CH-4), 15.40 (CH_3_); C_28_H_21_N_4_O_2_F; Calculated: C, 72.40; H, 4.56; N, 12.06. Found: C, 71.36; H, 4.32; N, 11.78.

#### 3.5.8. 2-Amino-6-(4-Ethoxyphenylazo)-4-(3-Nitro-Phenyl)-4H-Benzo[*h*]Chromene-3-Carbonitrile (4g)

Brown solid (88%), m.p. 216 °C; IR (KBr) cm^−1^: 3418, 3331 (NH_2_), 2980, 2967, 2923 (CH), 2210 (CN), 1571 (N = N), 1473, 1352 (NO_2_); ^1^H NMR (300 MHz, DMSO) δ 8.85–8.81 (m, 1H, Ar-H), 8.41–8.37 (m, 1H, Ar-H), 8.18 (s, 1H, Ar-H), 8.12 (d, *J* = 8.0 Hz, 1H, Ar-H), 7.92 (d, *J* = 8.9 Hz, 2H, Ar-H), 7.82–7.76 (m, 3H, Ar-H), 7.64 (d, *J* = 7.8 Hz, 1H, Ar-H), 7.45-7.41 (m, 3H, Ar-H, NH_2_), 7.09 (d, *J* = 8.9 Hz, 2H, Ar-H), 5.32 (s, 1H, H4), 4.12 (q, *J* = 6.8 Hz, 2H, CH_2_), 1.35 (t, *J* = 6.9 Hz, 3H, CH_3_); ^13^C NMR (75 MHz, DMSO) δ 162.33 (C-2), 160.83, 148.91, 148.58, 147.50, 145.91, 144.16 (Ar-C), 135.47, 131.47 (Ar-CH), 131.39 (Ar-C), 128.96, 128.40, 125.75 (Ar-CH), 124.23 (Ar-C), 124.02, 123.14, 122.98 (Ar-CH), 122.07 (Ar-C), 120.80 (Ar-CH), 117.99 (CN), 115.89, 112.43 (Ar-CH), 64.56 (CH_2_), 56.56 (C-3), 31.52 (CH-4), 15.38 (CH_3_); C_28_H_21_N_5_O_4_; Calculated: C, 68.42; H, 4.31; N, 14.25. Found: C, 69.33; H, 4.20; N, 13.46.

#### 3.5.9. Ethyl-2-Amino-6-(4-Ethoxyphenylazo)-4-(2-Bromo-Phenyl)-4H-Benzo[*h*]Chromene -3-Carboxylate (4h)

Dark brown solid (83%), m.p. 145.2 °C; IR (KBr) cm^−1^: 3451, 3296 (NH_2_), 2981, 2939, 2885 (CH), 1674 (CO), 1516 (N = N); ^1^H NMR (300 MHz, DMSO) δ 8.80 (dd, *J* = 5.9, 2.5 Hz, 1H, Ar-H), 8.42 (dd, *J* = 6.2, 3.4 Hz, 1H, Ar-H), 7.91 (m, 4H, Ar-H, NH_2_), 7.79–7.71 (m, 2H, Ar-H), 7.67 (s, 1H, Ar-H), 7.56 (d, *J* = 7.7 Hz, 1H, Ar-H), 7.27–7.19 (m, 2H, Ar-H), 7.11-7.05 (m, 3H, Ar-H), 5.64 (s, 1H, H4), 4.12 (q, *J* = 6.8 Hz, 2H, CH_2_), 3.51 (s, 3H, OCH_3_), 1.36 (t, *J* = 6.6 Hz, 3H, CH_3_); ^13^C NMR (75 MHz, DMSO) δ 169.22 (C=O), 168.92, 162.25 (Ar-C), 161.22 (C-2), 147.61, 147.47, 145.45 (Ar-C), 143.94, 133.50, 131.21, 129.24 (Ar-CH), 129.12 (Ar-C), 128.58, 128.15, 125.72 (Ar-CH), 124.20 (Ar-C), 123.16 (Ar-CH), 122.96 (Ar-C), 122.07 (Ar-CH), 120.94 (Ar-C), 115.89, 111.92 (Ar-CH), 76.29 (C-3), 64.55 (CH_2_), 51.36 (OCH_3_), 28.16 (CH-4), 15.38 (CH_3_); C_29_H_24_N_3_O_4_Br; Calculated: C, 62.94; H, 4.58; N, 7.34. Found: C, 63.75; H, 3.85; N, 8.10.

#### 3.5.10. Ethyl-2-Amino-6-(4-Ethoxyphenylazo)-4-(2-Chloro-Phenyl)-4H-Benzo[*h*]Chromene -3-Carboxylate (4i)

Light brown solid (81%), m.p. 139.9-140 °C; IR (KBr) cm^−1^: 3454, 3316 (NH_2_), 2977, 2934 (CH), 1675 (CO), 1535 (N = N); ^1^H NMR (300 MHz, DMSO) δ 8.79-8.76 (m, 1H, Ar-H), 8.41–8.37 (m, 1H, Ar-H), 7.93–7.86 (m, 4H, Ar-H, NH_2_), 7.75-7.67 (m, 2H, Ar-H), 7.56 (s, 1H, Ar-H), 7.37 (dd, *J* = 6.8, 1.10 Hz, 1H, Ar-H), 7.26 (ddd, *J* = 7.7, 3.7, 1.10 Hz, 1H, Ar-H), 7.20 (ddd, *J* = 6.6, 3.7, 1.7 Hz, 1H, Ar-H), 7.13 (dd, *J* = 7.6, 1.7 Hz, 1H, Ar-H), 7.05 (d, *J* = 9.0 Hz, 2H, Ar-H), 5.60 (s, 1H, H4), 4.08 (q, *J* = 6.9 Hz, 2H, CH_2_), 3.52 (s, 3H, OCH_3_), 1.33 (t, *J* = 6.9 Hz, 3H, CH_3_); ^13^C NMR (75 MHz, DMSO) δ 207.36 (C = O), 169.18, 168.88, 162.24 (Ar-C), 161.36 (C-2), 147.49, 145.54, 143.93 (Ar-C), 132.27, 131.24, 131.19 (Ar-CH), 130.33 (Ar-C), 128.88, 128.58, 128.16, 125.81 (Ar-CH), 124.14 (Ar-C), 123.89, 122.05 (Ar-CH), 120.71 (Ar-C), 115.89, 112.05 (Ar-CH), 75.97 (C-3), 64.55 (CH_2_), 51.38 (OCH_3_), 32.97 (CH-4), 15.38 (CH_3_); C_29_H_24_N_3_O_4_Cl; Calculated: C, 68.24; H, 4.96; N, 7.96. Found: C, 68.91; H, 4.20; N, 8.69.

#### 3.5.11. Ethyl-2-Amino-6-(4-Ethoxyphenylazo)-4-(3-Bromo-Phenyl)-4H-Benzo[*h*]Chromene-3-Carboxylate (4j)

Orange solid (86%), m.p. 158.9 °C; IR (KBr) cm^−1^: 3489, 3346 (NH_2_), 2976, 2949, 2871 (CH), 1675 (CO), 1487 (N = N); ^1^H NMR (300 MHz, DMSO) δ 8.82 (dd, *J* = 5.5, 3.1 Hz, 1H, Ar-H), 8.43 (dd, *J* = 6.4, 3.5 Hz, 1H, Ar-H), 7.95 (d, *J* = 8.9 Hz, 2H, Ar-H), 7.89 (s, 2H, NH_2_), 7.80–7.71 (m, 2H, Ar-H), 7.60 (s, 1H, Ar-H), 7.43 (s, 1H, Ar-H), 7.33-7.26 (m, 2H, Ar-H), 7.22-7.18 (m, 1H, Ar-H), 7.11 (d, *J* = 9.0 Hz, 2H, Ar-H), 5.16 (s, 1H, H4), 4.13 (q, *J* = 6.9 Hz, 2H, CH_2_), 3.57 (s, 3H, OCH_3_), 1.36 (t, *J* = 6.9 Hz, 3H, CH_3_); ^13^C NMR (75 MHz, DMSO) δ 169.05 (C = O), 162.25 (Ar-C), 161.31 (C-2), 151.13, 147.57, 145.96, 144.14 (Ar-C), 131.64 (Ar-CH), 131.12 (Ar-C), 130.73, 130.08, 128.60, 128.18, 127.35, 125.72 (Ar-CH), 124.19 (Ar-C), 123.97 (Ar-CH), 122.51 (Ar-C), 122.05 (Ar-CH), 121.46 (Ar-C), 115.88, 112.89 (Ar-CH), 79.93 (C-3), 78.32 (CH_2_), 57.67 (OCH_3_), 39.95 (CH-4), 18.45 (CH_3_); C_29_H_24_N_3_O_4_Br; Calculated: C, 62.94; H, 4.58; N, 7.34. Found: C, 63.20; H, 3.90; N, 7.74.

#### 3.5.12. Ethyl-2-Amino-6-(4-Ethoxyphenylazo)-4-(3-Nitro-Phenyl)-4H-Benzo[*h*]Chromene-3-Carboxylate (4k)

Orange solid (85%), m.p. 158.9 °C; IR (KBr) cm^−1^: 3489, 3346 (NH_2_), 2976, 2949, 2871 (CH), 1675 (CO), 1487 (N = N); ^1^H NMR (300 MHz, DMSO) δ 8.82 (dd, *J* = 5.5, 3.1 Hz, 1H, Ar-H), 8.43 (dd, *J* = 6.4, 3.5 Hz, 1H, Ar-H), 7.95 (d, *J* = 8.9 Hz, 2H, Ar-H), 7.89 (s, 2H, NH_2_), 7.80–7.71 (m, 2H, Ar-H), 7.60 (s, 1H, Ar-H), 7.43 (s, 1H, Ar-H), 7.33-7.26 (m, 2H, Ar-H), 7.22-7.18 (m, 1H, Ar-H), 7.11 (d, *J* = 9.0 Hz, 2H, Ar-H), 5.16 (s, 1H, H4), 4.13 (q, *J* = 6.9 Hz, 2H, CH_2_), 3.57 (s, 3H, OCH_3_), 1.36 (t, *J* = 6.9 Hz, 3H, CH_3_); ^13^C NMR (75 MHz, DMSO) δ 169.05 (C = O), 162.25 (Ar-C), 161.31 (C-2), 151.13, 147.57, 145.96, 144.14 (Ar-C), 131.64 (Ar-CH), 131.12 (Ar-C), 130.73, 130.08, 128.60, 128.18, 127.35, 125.72 (Ar-CH), 124.19 (Ar-C), 123.97 (Ar-CH), 122.51 (Ar-C), 122.05 (Ar-CH), 121.46 (Ar-C), 115.88, 112.89 (Ar-CH), 79.93 (C-3), 78.32 (CH_2_), 57.67 (OCH_3_), 39.95 (CH-4), 18.45 (CH_3_); C_29_H_24_N_3_O_4_Br; Calculated: C, 62.94; H, 4.58; N, 7.34. Found: C, 63.20; H, 3.90; N, 7.74.

#### 3.5.13. Ethyl-2-Amino-6-(4-Ethoxyphenylazo)-4-(4-Fluoro-Phenyl)-4H-Benzo[*h*]Chromene-3-Carboxylate (4l)

Greenish-brown solid (88%), m.p. 214 °C; IR (KBr) cm^−1^: 3466, 3278 (NH_2_), 2979, 2935, 2869 (CH), 2187 (CN), 1567 (N = N); ^1^H NMR (300 MHz, DMSO) δ 8.86–8.81 (m, 1H, Ar-H), 8.38–8.35 (m, 1H, Ar-H), 7.95 (d, *J* = 8.9 Hz, 2H, Ar-H), 7.81–7.76 (m, 2H, Ar-H), 7.41 (s, 1H, Ar-H), 7.35–7.27 (m, 4H, Ar-H, NH_2_), 7.19–7.09 (m, 4H, Ar-H), 5.07 (s, 1H, H4), 4.15 (d, *J* = 7.0 Hz, 2H, CH_2_), 1.37 (t, *J* = 6.9 Hz, 3H, CH_3_); ^13^C NMR (75 MHz, DMSO) δ 162.31(C-2), 160.51, 147.54, 145.74, 143.99, 142.69, 131.26 (Ar-C), 130.59, 130.48 (Ar-CH), 128.81 (Ar-C), 128.32, 125.76, 125.24 (Ar-CH), 124.21 (Ar-C), 123.98, 122.02 (Ar-CH), 120.99 (Ar-C), 118.92 (CN), 116.61, 116.32, 115.92, 112.60 (Ar-CH), 64.57 (CH_2_), 57.34 (C-3), 31.54 (CH-4), 15.40 (CH_3_); C_28_H_21_N_4_O_2_F; Calculated: C, 72.40; H, 4.56; N, 12.06. Found: C, 71.36; H, 4.32; N, 11.78.

## 4. Conclusions

This report discussed the synthesis of novel highly-colored chromene compounds incorporating azo chromophore moieties **4a-l** via the Knoevenagal condensation of the benzaldehyde derivatives and malononitrile or ethyl cyanoacetate, followed by the electrophilic substitution step of 1-naphthalenol-4-[(4-ethoxyphenyl) azo (Michael addition adduct). All the newly synthesized compounds were characterized, utilizing IR and NMR spectroscopy. The antimicrobial activity of the desired derivatives illustrated greater potency in appraisal with the reference drugs against certain fungal species and Gram-negative bacteria. On the other hand, these molecules, in evaluation with the same references, revealed comparable activity towards Gram-positive bacteria. Additionally, the novel compounds witnessed comparable or slightly lower cytotoxicity in comparison with the reference compounds. Overall, compounds **4l**, **4j**, and **4e** displayed good IC_50_, ranging from 4.35 to 5.54 µg/mL, against the HCT-116 with compounds **4l**, **4j**, and **4h,** exhibiting strong behavior against the MCF-7 cell lines. Moreover, all the target derivatives revealed less activity in the case of HepG-2 cell line except **4l**. The docking study results demonstrated the binding interactions of the synthesized compounds **4b** and **4h** in the active site of the *E. coli* DNA gyrase B enzyme. Lastly, the DFT results in terms of the structure–activity relationships (SAR) were also constructed to validate the obtained findings of the prepared compounds’ biological activity. Furthermore, the density distributions of the FMOs surfaces were employed to describe the molecules’ different biological activity profiles, where the active compounds HOMO’s character exhibited less density than their LUMO’s character, while the symmetrical distribution of these orbitals is related to lower biological activity.

## Figures and Tables

**Table 1:** The calculated energies of frontier molecular orbitals (FMOs), their energy gap (eV), and λ_max_ (nm) of the prepared compounds **4a-l**.

Compound	HOMO	LUMO	ΔE_LUMO-HOMO_	λ_max_ (Theo)
**4a**	−5.76	−2.51	3.25	381.95
**4b**	−5.78	−2.54	3.24	382.65
**4c**	−5.77	−2.53	3.24	382.97
**4d**	−5.83	−2.60	3.23	383.45
**4e**	−5.84	−2.61	3.23	383.42
**4f**	−5.83	−2.60	3.23	383.74
**4g**	−5.93	−3.01	2.92	424.23
**4h**	−5.65	−2.41	3.25	381.64
**4i**	−5.66	−2.41	3.25	381.54
**4j**	−5.73	−2.48	3.24	382.21
**4k**	−5.84	−2.90	2.94	421.93
**4l**	−5.60	−2.38	3.22	384.67

**Table 2:** Antimicrobial activity of the synthetic compounds (inhibition zone (IZ) diameter (mm)) (1 mg/mL).

Inhibition Zone Diameter (mm)								
Compounds	Gram Positive		Gram Negative		Fungi			
	S.P.	B.S.	P.A.	E.C.	A.F.	S.R.	G.C.	C.A.
**4a**	20.5 ± 0.44	22.4 ± 0.58	NA	19.4 ± 0.19	18.7 ± 0.25	15.6 ± 0.25	19.2 ± 0.58	NA
**4b**	18.4 ± 0.34	21.5 ± 0.25	NA	20.4 ± 0.58	20.9 ± 0.63	19.2 ± 0.27	22.8 ± 0.35	NA
**4c**	17.4 ± 0.34	20.2 ± 0.25	NA	17.7 ± 0.58	16.2 ± 0.63	17.1 ± 0.27	17.1 ± 0.35	NA
**4d**	19.6 ± 0.55	20.5 ± 0.52	NA	15.9 ± 0.58	18.6 ± 0.36	17.3 ± 0.35	18.9 ± 0.44	NA
**4e**	20.3 ± 0.44	20.9 ± 0.25	NA	15.4 ± 0.39	18.3 ± 0.55	20.1 ± 0.27	20.9 ± 0.58	NA
**4f**	20.3 ± 0.28	21.4 ± 0.37	NA	16.9 ± 0.19	17.6 ± 0.11	18.4 ± 0.23	20.9 ± 0.27	NA
**4g**	19.3 ± 0.16	20.3 ± 0.19	NA	18.9 ± 0.27	16.2 ± 0.20	20.8 ± 0.15	21.8 ± 0.18	NA
**4h**	19.6 ± 0.52	21.4 ± 0.63	NA	19.3 ± 0.62	19.3 ± 0.62	20.4 ± 0.34	20.8 ± 0.64	NA
**4i**	21.4 ± 0.25	23.4 ± 0.58	NA	21.3 ± 0.16	23.7 ± 0.26	21.4 ± 0.35	25.4 ± 0.18	NA
**4j**	16.1 ± 0.55	18.6 ± 0.25	NA	13.4 ± 0.19	10.6 ± 0.44	18.7 ± 0.25	23.4 ± 0.58	NA
**4k**	18.3 ± 0.19	20.3 ± 0.24	NA	17.7 ± 0.25	NA	15.4 ± 0.24	18.3 ± 0.15	NA
**4l**	20.8 ± 0.34	22.6 ± 0.25	NA	18.2 ± 0.58	18.7 ± 0.63	16.3 ± 0.27	19.4 ± 0.35	NA
**Ampicillin** **Gentamicin** **Amphotericin B**	21.9 ± 0.2	25.4 ± 0.3	–	–	–	–	–	–
	–	–	17.3 ± 0.1	19.9 ± 0.3	–	–	–	–
	–	–	–	–	23.9 ± 0.1	19.7 ± 0.2	17.9 ± 0.2	19.8 ± 0.1

Mean zone of inhibition in mm from at least three experiments; NA: no activity; S.P.: *Streptococcus pneumoniae*; B.S.: *Bacillus subtilis*; S.A.: *Staphylococcus aureus*; MRSA: Methicillin-resistant *Staphylococcus aureus*; P.A.: *Pseudomonas aeruginosa*; E.C.: *Escherichia coli*; S.T.: *Salmonella typhimurium*; A.F.: *Aspergillus fumigatus* (RCMB 02568); G.C.: *Geotricum candidum* (RCMB 05097); S.R. *Syncephalastrum racemosum* (RCMB 05922); and C.A.: *Candida albicans* (RCMB 05036).

**Table 3:** Antimicrobial activity of the synthetic compounds (minimum inhibitory concentration (MIC), µg/mL).

Minimal Inhibitory Concentration (MIC, µg/mL)								
Compounds	Gram Positive		Gram Negative		Fungi			
	S.P.	B.S.	P.A.	E.C.	A.F.	S.R.	G.C.	C.A.
**4a**	1.95	0.98	NA	62.5	15.63	31.25	7.81	NA
**4b**	7.81	1.95	NA	3.9	1.95	3.9	0.98	NA
**4c**	3.9	1.95	NA	31.25	7.81	15.63	0.98	NA
**4d**	3.9	15.63	NA	3.9	125	125	62.5	NA
**4e**	62.5	31.25	NA	15.63	125	125	62.5	NA
**4f**	7.81	7.81	NA	3.9	7.81	62.5	0.49	NA
**4g**	3.9	3.9	NA	3.9	31.25	3.9	1.95	NA
**4h**	0.98	1.95	NA	3.9	3.9	3.9	15.63	NA
**4i**	1.95	0.98	NA	1.95	0.98	1.95	0.49	NA
**4j**	7.81	7.81	NA	3.9	15.63	31.25	7.81	NA
**4k**	1.95	0.49	NA	15.63	NA	62.5	7.81	NA
**4l**	3.9	0.98	NA	7.81	7.81	31.25	3.9	NA
**Ampicillin** **Gentamicin** **Amphotericin B**	0.49	0.06	–	–	–	–	–	–
	–	–	3.9	7.81	–	–	–	–
	–	–	–	–	0.12	0.49	7.81	1.95

Mean zone of inhibition in mm from at least three experiments; NA: no activity, against four human pathogens and four fungi.

**Table 4:** Cytotoxicity of chromene derivatives against three different cancer cell lines.

Compounds	IC_50_ (µg/mL)		
	HCT-116	MCF-7	HepG-2
**4a**	42.1	21.9	11.6
**4b**	19.2	16.2	11.3
**4c**	12.2	21.1	7.28
**4d**	15.3	9.16	7.64
**4e**	5.54	19.7	9.99
**4f**	42.6	22.5	14.3
**4g**	9.01	7.14	12.0
**4h**	40.9	5.5	9.19
**4i**	30.0	11.5	8.76
**4j**	4.35	4.72	11.6
**4k**	11.2	9.5	10.5
**4l**	2.51	3.91	5.41
**Doxorubicin**	0.88	1.02	1.19
**Vinblastine**	2.6	6.1	4.6
**Colchicine**	42.8	17.7	10.6

**Table 5:** Description of the docking data of the selected target compounds.

Compound No.		4b	4h	Fat Brown B	1-(4-Ethoxyphenyl)-2-(4-Phenyl-4H-Benzo[*h*]Chromen-6-yl)Diazene
**Amino acid (** **Distance Å)**	**Asp 73**	-NH_2_ (2.3, 2.4)	–	--OH (2.4)	–
	**Thr 165**	-CN (3.2)	–	–	–
	**Asn 46**	–	--NH_2_ (2.0)	–	–
	**Asp 49**	–	--Br (3.2)	–	–
	**Ile 78**	–	-phenyl	–	–
**Interaction type**		H-bonding	H-bonding (aromatic)	H-bonding	H-bonding
***∆*** **G (kcal/mol) (MOE)**		−13.61	−12.23	−11.24	–
***∆*** **G (kcal/mol) (Pyrx)**		−9.0	−8.6	−7.7	−7.9

**Table 6:** Chemical reactivity descriptors and dipole moment (µ, Debye) of investigated compounds **4a-l**.

**Compounds**	$\chi =-\frac{1}{2}\left(E_{HOMO}+E_{LUMO}\right)$	$\eta =-\frac{1}{2}\left(E_{HOMO}-E_{LUMO}\right)$	$\delta =\frac{1}{\eta }\;\;$	$\omega =\frac{\chi^{2}}{2\eta }$	A = *−*E_LUMO_	I = *−*E_HOMO_	µ
**4a**	4.14	1.63	0.62	5.26	2.51	5.76	6.4
**4b**	4.16	1.62	0.62	5.34	2.54	5.78	6.9
**4c**	4.15	1.62	0.62	5.32	2.53	5.77	6.7
**4d**	4.22	1.62	0.62	5.50	2.60	5.83	7.7
**4e**	4.23	1.62	0.62	5.53	2.61	5.84	8.0
**4f**	4.22	1.62	0.62	5.50	2.60	5.83	7.2
**4g**	4.47	1.46	0.68	6.84	3.01	5.93	10.3
**4h**	4.03	1.62	0.62	5.01	2.41	5.65	2.9
**4i**	4.04	1.63	0.62	5.01	2.41	5.66	3.1
**4j**	4.11	1.63	0.62	5.18	2.48	5.73	4.2
**4k**	4.37	1.47	0.68	6.50	2.90	5.84	7.4
**4l**	3.99	1.61	0.62	4.94	2.38	5.60	2.5

**Table 7:** Lipophilicity, total polar surface area, H-bond acceptor, H-bond donor, violations, and volume of compounds **4a-l.**

Compound	Lipophilicity	Polar Surface Area	H-Bond Acceptor	H-Bond Donor	Violations	Volume
**4a**	6.57	93.01	6	2	1	407.77
**4b**	7.08	93.01	6	2	1	416.38
**4c**	7.21	93.01	6	2	2	420.73
**4d**	7.24	93.01	6	2	2	420.73
**4e**	7.11	93.01	6	2	1	416.38
**4f**	6.62	93.01	6	2	1	407.77
**4g**	6.62	93.01	6	2	1	407.77
**4h**	6.39	138.84	9	2	1	426.17
**4i**	7.67	95.52	7	2	2	465.20
**4j**	7.54	95.52	7	2	2	460.85
**4k**	7.69	95.52	7	2	2	465.20
**4l**	6.84	141.35	10	2	2	470.64

## Data Availability

Not applicable.

## References

[1] Gupta S.M., Mania-Pramanik J. (2019). RETRACTED ARTICLE: Molecular mechanisms in progression of HPV-associated cervical carcinogenesis. J. Biomed. Sci..

[2] Lee J., Pyo H.K., Park M.-O., Kim H., Yoon S.Y., Han J., Jung N., Yun S., Kim J., Chae Y.S. (2018). Top publications by reads. J. Stroke Cerebrovasc. Dis..

[3] Gourdeau H., Leblond L., Hamelin B., Desputeau C., Dong K., Kianicka I., Custeau D., Boudreau C., Geerts L., Cai S.-X. (2004). Antivascular and antitumor evaluation of 2-amino-4-(3-bromo-4, 5-dimethoxy-phenyl)-3-cyano-4H-chromenes, a novel series of anticancer agents. Mol. Cancer Ther..

[4] Sangani C.B., Shah N.M., Patel M.P., Patel R.G. (2012). Microwave assisted synthesis of novel 4H-chromene derivatives bearing phenoxypyrazole and their antimicrobial activity assess. J. Serb. Chem. Soc..

[5] Thareja S., Verma A., Kalra A., Gosain S., Rewatkar P.V., Kokil G.R. (2010). Novel chromeneimidazole derivatives as antifungal compounds: Synthesis and in vitro evaluation. Acta Pol. Pharm..

[6] Mori J., Iwashima M., Takeuchi M., Saito H. (2006). A synthetic study on antiviral and antioxidative chromene derivative. Chem. Pharm. Bull..

[7] Kamdar N.R., Haveliwala D.D., Mistry P.T., Patel S.K. (2011). Synthesis and evaluation of in vitro antitubercular activity and antimicrobial activity of some novel 4H-chromeno [2, 3-d] pyrimidine via 2-amino-4-phenyl-4H-chromene-3-carbonitriles. Med. Chem. Res..

[8] Gupta S., Kumar N., Kumar S., Dudhe R., Sharma P. (2012). 3-Hydroxy-2-(substituted phenyl)-4H-chromen-4-one derivatives-synthesis, spectral characterization and pharmacological screening. IJT A.

[9] Bhat M.A., Siddiqui N., Khan S.A. (2008). Synthesis of novel 3-(4-acetyl-5H/methyl-5-substituted phenyl-4, 5-dihydro-1, 3, 4-oxadiazol-2-yl)-2H-chromen-2-ones as potential anticonvulsant agents. Acta Pol. Pharm..

[10] Venugopala K.N., Rashmi V., Odhav B. (2013). Review on natural coumarin lead compounds for their pharmacological activity. BioMed Res. Int..

[11] Mccarroll J., Parker A., Kavallaris M. (2014). Microtubules and their role in cellular stress in cancer. Front. Oncol..

[12] Ren Q., Siau W.Y., Du Z., Zhang K., Wang J. (2011). Expeditious assembly of a 2-amino-4H-chromene skeleton by using an enantioselective mannich intramolecular ring cyclization–tautomerization cascade sequence. Chem. A Eur. J..

[13] Jain N., Xu J., Kanojia R.M., Du F., Jian-Zhong G., Pacia E., Lai M.-T., Musto A., Allan G., Reuman M. (2009). Identification and Structure–Activity Relationships of Chromene-Derived Selective Estrogen Receptor Modulators for Treatment of Postmenopausal Symptoms. J. Med. Chem..

[14] Alblewi F.F., Okasha R.M., Eskandrani A.A., Afifi T.H., Mohamed H.M., Halawa A.H., Fouda A.M., Al-Dies A.-A.M., Mora A., El-Agrody A.M. (2019). Design and synthesis of novel heterocyclic-based 4H-benzo [*h*] chromene moieties: Targeting antitumor caspase 3/7 activities and cell cycle analysis. Molecules.

[15] Ahmed H.E., El-Nassag M.A., Hassan A.H., Mohamed H.M., Halawa A.H., Okasha R.M., Ihmaid S., Abd El-Gilil S.M., Khattab E.S., Fouda A.M. (2019). Developing lipophilic aromatic halogenated fused systems with specific ring orientations, leading to potent anticancer analogs and targeting the c-Src Kinase enzyme. J. Mol. Struct..

[16] Alblewi F.F., Okasha R.M., Hritani Z.M., Mohamed H.M., El-Nassag M.A., Halawa A.H., Mora A., Fouda A.M., Assiri M.A., Al-Dies A.-A.M. (2019). Antiproliferative effect, cell cycle arrest and apoptosis generation of novel synthesized anticancer heterocyclic derivatives based 4H-benzo [*h*] chromene. Bioorg. Chem..

[17] Okasha R.M., Alblewi F.F., Afifi T.H., Naqvi A., Fouda A.M., Al-Dies A.-A.M., El-Agrody A.M. (2017). Design of new benzo [*h*] chromene derivatives: Antitumor activities and structure-activity relationships of the 2, 3-positions and fused rings at the 2, 3-positions. Molecules.

[18] Abd-El-Aziz A., El-Ghezlani E., Elaasser M., Afifi T., Okasha R. (2020). First Example of Cationic Cyclopentadienyliron Based Chromene Complexes and Polymers: Synthesis, Characterization, and Biological Applications. J. Inorg. Organomet. Polym. Mater..

[19] Khafagy M.M., Abd El-Wahab A.H., Eid F.A., El-Agrody A.M. (2002). Synthesis of halogen derivatives of benzo [*h*] chromene and benzo [a] anthracene with promising antimicrobial activities. Il Farmaco.

[20] Smith P.W., Sollis S.L., Howes P.D., Cherry P.C., Starkey I.D., Cobley K.N., Weston H., Scicinski J., Merritt A., Whittington A. (1998). Dihydropyrancarboxamides related to zanamivir: A new series of inhibitors of influenza virus sialidases. 1. Discovery, synthesis, biological activity, and structure− activity relationships of 4-guanidino-and 4-amino-4 H-pyran-6-carboxamides. J. Med. Chem..

[21] Naseer M.A., Husain A. (2019). Studies on Chromene based 2, 6-disubstituted-Thiazolo [3, 2-B][1, 2, 4] Triazole derivatives: Synthesis and Biological Evaluation. J. Drug Deliv. Ther..

[22] Safari F., Hosseini H., Bayat M., Ranjbar A. (2019). Synthesis and evaluation of antimicrobial activity, cytotoxic and pro-apoptotic effects of novel spiro-4 H-pyran derivatives. RSC Adv..

[23] El-Agrody A.M., Al-Dies A.-A.M., Fouda A.M. (2014). Microwave assisted synthesis of 2-amino-6-methoxy-4H-benzo [*h*] chromene derivatives. Eur. J. Chem..

[24] Sabry N.M., Mohamed H.M., Khattab E.S.A., Motlaq S.S., El-Agrody A.M. (2011). Synthesis of 4H-chromene, coumarin, 12H-chromeno [2, 3-d] pyrimidine derivatives and some of their antimicrobial and cytotoxicity activities. Eur. J. Med. Chem..

[25] Kushwaha R.K., Singh K., Kumar P., Chandra D. (2019). Review on Chromen derivatives and their Pharmacological Activities. Res. J. Pharm. Technol..

[26] Halawa A.H., Elaasser M.M., El Kerdawy A.M., Abd El-Hady A.M., Emam H.A., El-Agrody A.M. (2017). Anticancer activities, molecular docking and structure–activity relationship of novel synthesized 4H-chromene, and 5H-chromeno [2, 3-d] pyrimidine candidates. Med. Chem. Res..

[27] Anderson R., Nickless G. (1967). Heterocyclic azo dyestuffs in analytical chemistry. A review. Analyst.

[28] Chung K.-T. (2016). Azo dyes and human health: A review. J. Environ. Sci. Health Part C.

[29] Rafii F., Hall J., Cerniglia C. (1997). Mutagenicity of azo dyes used in foods, drugs and cosmetics before and after reduction by Clostridium species from the human intestinal tract. Food Chem. Toxicol..

[30] Alhaddad O.A., Abu Al-Ola K.A., Hagar M., Ahmed H.A. (2020). Chair-and V-Shaped of H-bonded Supramolecular Complexes of Azophenyl Nicotinate Derivatives; Mesomorphic and DFT Molecular Geometry Aspects. Molecules.

[31] Ahmed N.H., Saad G.R., Ahmed H.A., Hagar M. (2020). New wide-stability four-ring azo/ester/Schiff base liquid crystals: Synthesis, mesomorphic, photophysical, and DFT approaches. RSC Adv..

[32] Alhaddad O.A., Ahmed H.A., Hagar M., Saad G.R., Abu Al-Ola K.A., Naoum M.M. (2020). Thermal and photophysical studies of binary mixtures of liquid crystal with different geometrical mesogens. Crystals.

[33] Al-Mutabagani L.A., Abdullah Alshabanah L., Ahmed H.A., Abu Al-Ola K.A., Hagar M. (2020). New Rod-like H-bonded Assembly Systems: Mesomorphic and Geometrical Aspects. Crystals.

[34] Weglarz-Tomczak E., Gorecki L. (2012). Azo dyes–Biological activity and synthetic strategy. Chemik.

[35] Thaokar S.F., Patel D.M., Patel M.P., Patel R.G. (2007). Synthesis and antibacterial activity of novel pyrazolo [3, 4-b] quinoline based heterocyclic azo compounds and their dyeing performance. Saudi Pharm. J..

[36] Parekh N., Maheria K., Patel P., Rathod M. (2011). Study on antibacterial activity for multidrug resistance stain by using phenyl pyrazolones substituted 3-amino 1H-pyrazolon (3, 4-b) quinoline derivative in vitro condition. Int. J. Pharm. Tech. Res..

[37] Khedr A.M., Gaber M., Abd El-Zaher E.H. (2011). Synthesis, structural characterization, and antimicrobial activities of Mn (II), Co (II), Ni (II), Cu (II) and Zn (II) complexes of triazole-based azodyes. Chin. J. Chem..

[38] Swati G., Romila K., Sharma I., Verma P. (2011). Synthesis, characterization and antimicrobial screening of some azo compounds. Int. J. Appl. Biol. Pharm. Tech..

[39] Kantar C., Akal H., Kaya B., Islamoğlu F., Türk M., Şaşmaz S. (2015). Novel phthalocyanines containing resorcinol azo dyes; synthesis, determination of pKa values, antioxidant, antibacterial and anticancer activity. J. Organomet. Chem..

[40] Abadi A.H., Eissa A.A.H., Hassan G.S. (2003). Synthesis of novel 1, 3, 4-trisubstituted pyrazole derivatives and their evaluation as antitumor and antiangiogenic agents. Chem. Pharm. Bull..

[41] Afifi T.H., Okasha R.M., Alsherif H., Ahmed H.E.A., Abd-El-Aziz A.S. (2017). Design, synthesis, and docking studies of 4H-chromene and chromene based azo chromophores: A novel series of potent antimicrobial and anticancer agents. Curr. Org. Synth..

[42] Afifi T.H., Okasha R.M., Ahmed H.E., Ilaš J., Saleh T., Abd-El-Aziz A.S. (2017). Structure-activity relationships and molecular docking studies of chromene and chromene based azo chromophores: A novel series of potent antimicrobial and anticancer agents. Excli J..

[43] Abd-El-Aziz A.S., Alsaggaf A.T., Okasha R.M., Ahmed H.E., Bissessur R., Abdelghani A.A., Afifi T.H. (2016). Antimicrobial and Antitumor Screening of Fluorescent 5, 7-Dihydroxy-4-Propyl-2H-Chromen-2-One Derivatives with Docking Studies. ChemistrySelect.

[44] Ali A.A.S., Khan D., Naqvi A., Al-Blewi F.F., Rezki N., Aouad M.R., Hagar M. (2021). Design, Synthesis, Molecular Modeling, Anticancer Studies, and Density Functional Theory Calculations of 4-(1, 2, 4-Triazol-3-ylsulfanylmethyl)-1, 2, 3-triazole Derivatives. ACS Omega.

[45] Mohammed F.F., Hagar M., Parveen S., Alnoman R.B., Ahmed H.A., Ashry E.S.H.E., Rasheed H.A. (2021). 2-(Alkylthio)-3-(Naphthalen-1-yl) Quinazolin-4 (3 H)-Ones: Ultrasonic Synthesis, DFT and Molecular Docking Aspects. Polycycl. Aromat. Compd..

[46] Parveen S., Hagar M.B., Alnoman R., Ahmed H.A., El Ashry E.S.H., Zakaria M.A. (2021). Synthesis, Docking and Density Functional Theory Approaches on 1, 3-Bis-3-(4-Chlorophenyl)-2, 3-Dihydroquinazolin-4 (1H)-on-2-Thioxopropane toward the Discovery of Dual Kinase Inhibitor. Polycycl. Aromat. Compd..

[47] Voskressensky L.G., Festa A.A., Varlamov A.V. (2014). Domino reactions based on Knoevenagel condensation in the synthesis of heterocyclic compounds. Recent advances. Tetrahedron.

[48] Griffiths J. (1981). Recent developments in the color and constitution of organic dyes. Rev. Prog. Coloration Relat. Top..

[49] EUCAST (2000). European Committee for Antimicrobial Susceptibility Testing (EUCAST) of the European Society of Clinical Microbiology and Infectious Diseases (ESCMID): Terminology relating to methods for the determination of susceptibility of bacteria to antimicrobial agents. CMI.

[50] Dabur R., Chhillar A., Yadav V., Kamal P.K., Gupta J., Sharma G. (2005). In vitro antifungal activity of 2-(3, 4-dimethyl-2, 5-dihydro-1H-pyrrol-2-yl)-1-methylethyl pentanoate, a dihydropyrrole derivative. J. Med. Microbiol..

[51] Choudhary M.I., Thomsen W.J. (2001). Bioassay Techniques for Drug Development.

[52] Mosmann T. (1983). Rapid colorimetric assay for cellular growth and survival: Application to proliferation and cytotoxicity assays. J. Immunol. Methods.

[53] Alley M.C., Scudiero D.A., Monks A., Hursey M.L., Czerwinski M.J., Fine D.L., Abbott B.J., Mayo J.G., Shoemaker R.H., Boyd M.R. (1988). Feasibility of drug screening with panels of human tumor cell lines using a microculture tetrazolium assay. Cancer Res..

[54] Alaa A.-M., Asiri Y.A., Al-Agamy M.H. (2011). Design, synthesis and antibacterial activity of fluoroquinolones containing bulky arenesulfonyl fragment: 2D-QSAR and docking study. Eur. J. Med. Chem..

[55] Trott O., Olson A.J. (2010). AutoDock Vina: Improving the speed and accuracy of docking with a new scoring function, efficient optimization, and multithreading. J. Comput. Chem..

[56] Ahmed H.E., El-Nassag M.A., Hassan A.H., Okasha R.M., Ihmaid S., Fouda A.M., Afifi T.H., Aljuhani A., El-Agrody A.M. (2018). Introducing novel potent anticancer agents of 1H-benzo [f] chromene scaffolds, targeting c-Src kinase enzyme with MDA-MB-231 cell line anti-invasion effect. J. Enzym. Inhib. Med. Chem..

[57] Hagar M., Ahmed H.A., Aljohani G., Alhaddad O.A. (2020). Investigation of Some Antiviral N-Heterocycles as COVID 19 Drug: Molecular Docking and DFT Calculations. Int. J. Mol. Sci..

[58] Al-Otaibi J.S., Mary Y.S., Mary Y.S., Panicker C.Y., Thomas R. (2019). Cocrystals of pyrazinamide with p-toluenesulfonic and ferulic acids: DFT investigations and molecular docking studies. J. Mol. Struct..

[59] Mohapatra R.K., El-ajaily M.M., Alassbaly F.S., Sarangi A.K., Das D., Maihub A.A., Ben-Gweirif S.F., Mahal A., Suleiman M., Perekhoda L. (2020). DFT, anticancer, antioxidant and molecular docking investigations of some ternary Ni (II) complexes with 2-[(E)-[4-(dimethylamino) phenyl] methyleneamino] phenol. Chem. Pap..

[60] Joshi R., Pandey N., Yadav S.K., Tilak R., Mishra H., Pokharia S. (2018). Synthesis, spectroscopic characterization, DFT studies and antifungal activity of (E)-4-amino-5-[N’-(2-nitro-benzylidene)-hydrazino]-2, 4-dihydro-[1, 2, 4] triazole-3-thione. J. Mol. Struct..

[61] Joshi R., Kumari A., Singh K., Mishra H., Pokharia S. (2020). Triorganotin (IV) complexes of Schiff base derived from 1, 2, 4-triazole moiety: Synthesis, spectroscopic investigation, DFT studies, antifungal activity and molecular docking studies. J. Mol. Struct..

[62] Khodair A.I., Awad M.K., Gesson J.-P., Elshaier Y.A. (2020). New N-ribosides and N-mannosides of rhodanine derivatives with anticancer activity on leukemia cell line: Design, synthesis, DFT and molecular modelling studies. Carbohydr. Res..

[63] Kumar S.S., Athimoolam S., Sridhar B. (2018). Structural, spectral, theoretical and anticancer studies on new co-crystal of the drug 5-fluorouracil. J. Mol. Struct..

[64] Hagar M., Ahmed H., El-Sayed T., Alnoman R. (2019). Mesophase behavior and DFT conformational analysis of new symmetrical diester chalcone liquid crystals. J. Mol. Liq..

[65] Grover M., Singh B., Bakshi M., Singh S. (2000). Quantitative structure–property relationships in pharmaceutical research–Part 1. Pharm. Sci. Technol. Today.

[66] Malhotra R., Ravesh A., Singh V. (2017). Synthesis, characterization, antimicrobial activities, and QSAR studies of organotin (IV) complexes. Phosphorus Sulfur Silicon Relat. Elem..

[67] Kumer A., Sarker M.N., Paul S. (2019). The Simulating Study of HOMO, LUMO, thermo physical and Quantitative Structure of Activity Relationship (QSAR) of Some Anticancer Active Ionic Liquids. Eurasian J. Environ. Res..

[68] Hagar M., Chaieb K., Parveen S., Ahmed H., Alnoman R. (2020). N-alkyl 2-pyridone versus O-alkyl 2-pyridol: Ultrasonic synthesis, DFT, docking studies and their antimicrobial evaluation. J. Mol. Struct..

[69] Ali M.S., Farah M.A., Al-Lohedan H.A., Al-Anazi K.M. (2018). Comprehensive exploration of the anticancer activities of procaine and its binding with calf thymus DNA: A multi spectroscopic and molecular modelling study. RSC Adv..

[70] Rachedi K.O., Ouk T.-S., Bahadi R., Bouzina A., Djouad S.-E., Bechlem K., Zerrouki R., Hadda T.B., Almalki F., Berredjem M. (2019). Synthesis, DFT and POM analyses of cytotoxicity activity of α-amidophosphonates derivatives: Identification of potential antiviral O, O-pharmacophore site. J. Mol. Struct..

[71] Da Costa R.M., Bastos J.K., Costa M.C., Ferreira M.M., Mizuno C.S., Caramori G.F., Nagurniak G.R., Simão M.R., Dos Santos R.A., Veneziani R.C. (2018). In vitro cytotoxicity and structure-activity relationship approaches of ent-kaurenoic acid derivatives against human breast carcinoma cell line. Phytochemistry.

[72] Lewis D.F. (2003). Quantitative structure–activity relationships (QSARs) within the cytochrome P450 system: QSARs describing substrate binding, inhibition and induction of P450s. Inflammopharmacology.

[73] Almehmadi M.A., Aljuhani A., Alraqa S.Y., Ali I., Rezki N., Aouad M.R., Hagar M. (2021). Design, synthesis, DNA binding, modeling, anticancer studies and DFT calculations of Schiff bases tethering benzothiazole-1, 2, 3-triazole conjugates. J. Mol. Struct..

[74] Bouachrine M., Hamidi M., BOUZZINEA S., Taoufik H. (2009). Theoretical study on the structure and electronic properties of new materials based on thiophene and oxadiazole. J. Appl. Chem. Res..

[75] Yang L., Feng J.-K., Ren A.-M. (2005). Theoretical studies on the electronic and optical properties of two thiophene–fluorene based π-conjugated copolymers. Polymer.

[76] Alnoman R.B., Parveen S., Hagar M., Ahmed H.A., Knight J.G. (2019). A new chiral boron-dipyrromethene (BODIPY)-based fluorescent probe: Molecular docking, DFT, antibacterial and antioxidant approaches. J. Biomol. Struct. Dyn..

[77] Alnoman R.B., Hagar M., Parveen S., Ahmed H.A., Knight J.G. (2020). Computational and molecular docking approaches of a New axially chiral BODIPY fluorescent dye. J. Photochem. Photobiol. A Chem..

[78] Kouza M., Banerji A., Kolinski A., Buhimschi I., Kloczkowski A. (2018). Role of Resultant Dipole Moment in Mechanical Dissociation of Biological Complexes. Molecules.

[79] Shawon J., Khan A.M., Rahman A., Hoque M.M., Khan M.A.K., Sarwar M.G., Halim M.A. (2018). Molecular Recognition of Azelaic Acid and Related Molecules with DNA Polymerase I Investigated by Molecular Modeling Calculations. Interdiscip. Sci. Comput. Life Sci..

[80] Uzzaman M., Hoque M.J. (2018). Physiochemical, molecular docking, and pharmacokinetic studies of Naproxen and its modified derivatives based on DFT. Int. J. Sci. Res. Manag..

[81] Ortega J.T., Serrano M.L., Pujol F.H., Rangel H.R. (2020). Unrevealing sequence and structural features of novel coronavirus using in silico approaches: The main protease as molecular target. EXCLI J..

[82] Gangadevi V., Muthumary J. (2008). Isolation of Colletotrichum gloeosporioides, a novel endophytic taxol-producing fungus from the leaves of a medicinal plant, Justicia gendarussa. Mycol. Balc..

[83] Gangadevi V., Muthumary J. (2007). Preliminary studies on cytotoxic effect of fungal taxol on cancer cell lines. Afr. J. Biotechnol..

[84] Chemical Computing Group Inc (2016). Molecular Operating Environment (MOE).

